# Mitochondrial genomes reveal an east to west cline of steppe ancestry in Corded Ware populations

**DOI:** 10.1038/s41598-018-29914-5

**Published:** 2018-08-02

**Authors:** Anna Juras, Maciej Chyleński, Edvard Ehler, Helena Malmström, Danuta Żurkiewicz, Piotr Włodarczak, Stanisław Wilk, Jaroslav Peška, Pavel Fojtík, Miroslav Králík, Jerzy Libera, Jolanta Bagińska, Krzysztof Tunia, Viktor I. Klochko, Miroslawa Dabert, Mattias Jakobsson, Aleksander Kośko

**Affiliations:** 1 Department of Human Evolutionary Biology, Institute of Anthropology, Faculty of Biology, Adam Mickiewicz University in Poznan, Umultowska 89, 61-614 Poznań, Poland; 2 Institute of Archaeology, Faculty of History, Adam Mickiewicz University in Poznan, Umultowska 89D, 61-614 Poznań, Poland; 3 Laboratory of Genomics and Bioinformatics, Institute of Molecular Genetics of the ASCR, v. v. i., Vídeňská 1083, 142 20 Prague 4, Czech Republic; 4 Human Evolution, Department of Organismal Biology and SciLifeLab, Uppsala University, Norbyvägen 18C, SE-752 36 Uppsala, Sweden; 5 Polish Academy of Sciences, Institute of Archaeology and Ethnology, Sławkowska str. 17, 31-016 Kraków, Poland; 6 Institute of Archaeology, Jagiellonian University, Gołębia 11, 31-007 Kraków, Poland; 7 Archaeological Centre Olomouc, U Hradiska 42/6, 779 00 Olomouc, Czech Republic; 8 Department of History - Section of Archaeology, Philosophical faculty, Palacký University Olomouc, Na Hradě 5, 771 80 Olomouc, Czech Republic; 9 Institute of Archaeological Heritage Brno, v.v.i., Kaloudova 30, 614 00 Brno, Czech Republic; 10 Laboratory of Morphology and Forensic Anthropology (LaMorFA), Department of Anthropology, Faculty of Science, Masaryk University, Kotlářská 267/2, 611 37 Brno, Czech Republic; 11 Institute of Archaeology, Maria Curie-Skłodowska University, Maria Curie-Skłodowska Square 4, 20-031 Lublin, Poland; 12 Muzeum Regionalne im. Janusza Petera, ul. Zamojska 2, 22-600 Tomaszów Lubelski, Poland; 13 National University of “Kyiv-Mohyla Academy”, Institute of Archaeology, Hryhoriya Skovorody St. 2, 04655 Kyiv, Ukraine; 14 Molecular Biology Techniques Laboratory, Faculty of Biology, Adam Mickiewicz University in Poznan, Umultowska 89, 61-614 Poznań, Poland; 15 Centre for Anthropological Research, University of Johannesburg, Auckland Park, 2006 Johannesburg, South Africa

## Abstract

From around 4,000 to 2,000 BC the forest-steppe north-western Pontic region was occupied by people who shared a nomadic lifestyle, pastoral economy and barrow burial rituals. It has been shown that these groups, especially those associated with the Yamnaya culture, played an important role in shaping the gene pool of Bronze Age Europeans, which extends into present-day patterns of genetic variation in Europe. Although the genetic impact of these migrations from the forest-steppe Pontic region into central Europe have previously been addressed in several studies, the contribution of mitochondrial lineages to the people associated with the Corded Ware culture in the eastern part of the North European Plain remains contentious. In this study, we present mitochondrial genomes from 23 Late Eneolithic and Bronze Age individuals, including representatives of the north-western Pontic region and the Corded Ware culture from the eastern part of the North European Plain. We identified, for the first time in ancient populations, the rare mitochondrial haplogroup X4 in two Bronze Age Catacomb culture-associated individuals. Genetic similarity analyses show close maternal genetic affinities between populations associated with both eastern and Baltic Corded Ware culture, and the Yamnaya horizon, in contrast to larger genetic differentiation between populations associated with western Corded Ware culture and the Yamnaya horizon. This indicates that females with steppe ancestry contributed to the formation of populations associated with the eastern Corded Ware culture while more local people, likely of Neolithic farmer ancestry, contributed to the formation of populations associated with western Corded Ware culture.

## Introduction

The forest-steppe north-western Pontic region of the middle Dniester and Prut interfluve was a place of contact and exchange routes between human populations inhabiting the drainages of the Black and Baltic Seas from around 4,000 to 2,000 BC^1^. During this time, the region was occupied by forest-steppe populations attributed to the Eneolithic (3350–3200 BC)^1^ and the succeeding Bronze Age groups associated with the Yamnaya - Pit Grave (dated to 3,100/3,050–2,800 BC)^2^, the Catacomb (2,600–2,200 BC), the Babyno (2,200–1,700/1,600 BC) and the Noua (1,600–1,200/1,100 BC) cultures^3,4^. Although there were cultural differences between these populations, they all shared a similar nomadic lifestyle, pastoral economy and barrow burial rituals^5^. Some of the rounded burial mounds founded by Eneolithic people were reused by the succeeding cultural entities of the Early Bronze Age^1^, while other kurgans shared a mix of characteristics from both the Late Eneolithic and the Early Bronze Age funeral rites^1,4^.

According to some researchers^6–9^, the Yamnaya culture originated in the Volga-Ural interfluve and spread across the Pontic-Caspian steppe between 3,300–2,800 BC. This cultural expansion led to the development of a less homogenous group of cultural entities belonging to the so-called Yamnaya Cultural-Historical Area/Unity^10,11^, hereafter reffered to as ‘the Yamnaya horizon’^12^. People associated with the eastern Yamnaya culture spread across the steppe to the east of Don River. With no settlements identified in this area, they were thought to be more mobile because of their supposed nomadic profile of economy stimulated by environmental conditions of Kuban – North Caspian steppes^13^. On the other hand, Yamnaya settlements were found more frequently in the forest-steppe Pontic regions, to the west of Don River, probably due to favorable environmental conditions^12^.

One of the most widely debated issues, which emerged in connection to studies on the Yamnaya horizon, was the relationship between the people associated with the Yamnaya and the Central European final Neolithic cultures, in particular the Corded Ware culture (dated to 2800–2300 BC)^14^. Archaeological records point to some similarities between the Corded Ware culture and the steppe, including shared practices such as the barrow structures and burial rituals^2^. Adoption of a herding economy based on mobility through the use of wagons and horses, was also proposed as a common trait associated with both the Yamnaya and Corded Ware cultures^12^. These observations led some researchers to suggest a possible Yamnaya migration toward the Baltic drainage basin^15^ or a massive westward expansion of the steppe pastoralist people, representing the “barrow culture”, into the North European Plain^12,16^. However, specific burial customs of the Yamnaya people, such as the scarcity of grave goods, the presence of ochre, and the building of specific wooden roof or floor structures, generated opposing arguments emphasizing the significant differences between the Corded Ware and steppe cultures^2^.

Recent ancient DNA (aDNA) studies suggest that the large-scale migration of steppe populations associated with the Yamnaya horizon contributed to the formation of the final Neolithic central European populations^14,17,18^. Moreover, people associated with the Yamnaya horizon have been shown to be an admixed population with ancestry from Eastern hunter-gatherers and Caucasus hunter-gatherers^14,17,19,20^. Ancient DNA data indicate that the Neolithic populations from Central Europe already had the ‘Caucasus’ genetic component from the eastern steppes around 2,500 BC. Presence of this genetic component was used as an argument for the expansion of people from the Pontic-Caspian region into the central Europe^14,17^. X chromosome sequence data suggest that it was primarily males who participated in these migrations^21,22^ and contributed to the formation of the people associated with the Corded Ware culture^14,17^. Based on the X chromosome data obtained mainly from western Corded Ware-associated individuals, it was estimated that, for every female, ~4–15 males migrated from the steppe^21^. Subsequently, the Yamnaya genetic component spread across Bronze Age Europe and West Asia^14^.

Although questions concerning the migrations of nomadic people have been addressed by a number of studies^19,23,24^, the contribution of mitochondrial lineages associated with the Yamnaya horizon to the formation of people associated with the Corded Ware culture from the eastern part of the North European Plain, especially from the region of modern Poland, remains contentious. To investigate the maternal relationship between these two groups, we generated complete mitochondrial genomes from the representatives of Late Eneolithic and Early Bronze Age populations from the north-western Pontic region, including Yamnaya groups and individuals associated with the Corded Ware culture from the eastern part of the North European Plain.

## Materials and Methods

### Bone samples

**Figure 1:**
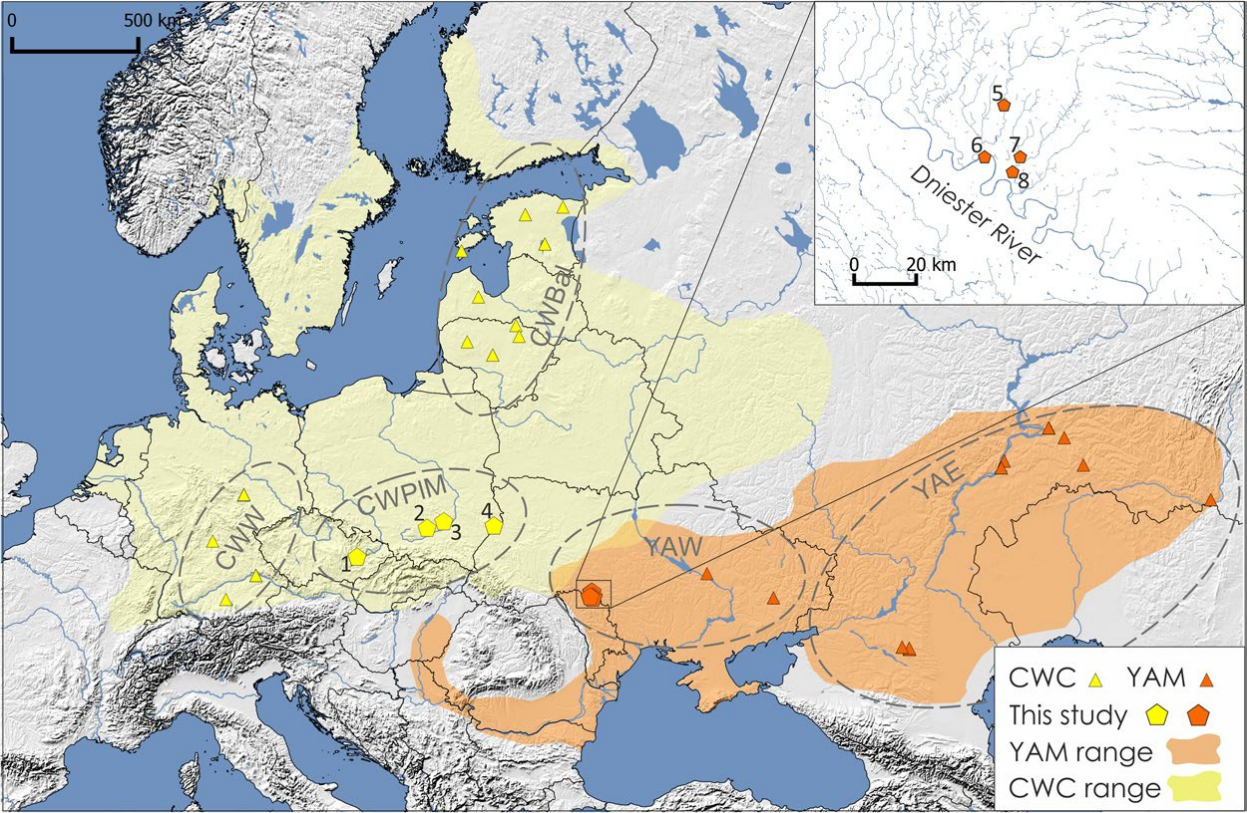
Location of archaeological sites used in this study. Extent of the Corded Ware culture (CWC) region and the Yamnaya horizon (YAM) are marked in yellow and orange, respectively. Groups of Corded Ware culture, including western (CWW), eastern (CWPlM) and Baltic (CWBal), and groups of Yamnaya horizon including western (YAW) and eastern (YAE), are marked in circles. Numbers represent archaeological sites from which analyzed individuals came from: (1) Držovice; (2) Malżyce; (3) Książnice; (4) Hubinek; (5) Klembivka; (6) Porohy; (7) Pidlisivka; (8) Prydnistryanske. The map was created using QGIS 2.12.2^47^.

Ancient DNA was extracted from the Corded Ware culture individuals excavated in south-eastern Poland (N = 12) and Moravia (N = 3). Late Eneolithic (N = 5) and Bronze Age human remains (N = 25) originated from western Ukraine and came from the Yampil barrow cemetery complex located in the north–western region of the Black Sea. Bronze Age individuals were associated with different archaeological cultures, including Yamnaya (N = 14), Catacomb (N = 2), Babyno (N = 4) and Noua (N = 5). The sampling localities are shown in Fig. 1. Detailed information about sampled individuals can be found in Table 1, Supplementary Information Text (Materials) and Supplementary Table S1.

**Table 1:** Description of analyzed individuals.

Sample ID	Region	Archaeol. site	Archaeol. culture	Age of samples	Molecular sex	MtDNA genome coverage	MtDNA haplogroup
poz090	Ukraine	Pidlisivka	Late Eneolithic	*3350–3200 BC*	XY	6x	U2e1a1
poz094	Ukraine	Pidlisivka	Babyno	*2200–1700/1600 BC*	XX	194x	J2b1a
poz211	Ukraine	Klembivka	Late Eneolithic	2898–2761 BC	XY	11x	U5a2b
poz213	Ukraine	Klembivka	Babyno	2117–1950 BC	XX	32x	J1c2m
poz214	Ukraine	Klembivka	Late Eneolithic	2863–2630 BC	XY	58x	H2a1
poz356	Ukraine	Klembivka	Babyno	1880–1771 BC	XX	40x	H1e
poz220	Ukraine	Prydnistryanske	Catacomb	2834–2499 BC	XY	27x	X4
poz221	Ukraine	Prydnistryanske	Catacomb	2548–2348 BC	XY	114x	X4
poz222	Ukraine	Prydnistryanske	Yamnaya	3023–2911 BC	XY	15.6x	W3a1
poz225	Ukraine	Prydnistryanske	Yamnaya	2858–2621 BC	n.a.	73x	U5a1i1
poz224	Ukraine	Prydnistryanske	Yamnaya	2847–2574 BC	n.a.	97.5x	U4c1
poz208	Ukraine	Porohy	Yamnaya	2882–2698 BC	n.a.	42x	W3a1a
poz287	Poland	Książnice	Corded Ware	*2400–2300 BC*	XX	28x	H6a
poz286	Poland	Książnice	Corded Ware	*2400–2300 BC*	XX	19x	H15a1
poz256	Moravia	Držovice	Corded Ware	*2800–2300 BC*	n.a.	71x	U4b1a1a
poz257	Moravia	Držovice	Corded Ware	*2800–2300 BC*	XX	64x	I4a
poz235	Poland	Hubinek	Corded Ware	*2800–2300 BC*	n.a.	5.4x	H2a2
poz279	Poland	Malżyce	Corded Ware	2454–2236 BC	n.a.	12x	W5b
poz280	Poland	Malżyce	Corded Ware	*2800–2300 BC*	n.a.	57x	W5b
poz281	Poland	Malżyce	Corded Ware	*2800–2300 BC*	n.a	79x	T2e
poz282	Poland	Malżyce	Corded Ware	*2800–2300 BC*	n.a.	36x	U4a2f
poz234	Poland	Hubinek	Corded Ware	*2800–2300 BC*	n.a.	39x	H1e
poz232	Poland	Hubinek	Corded Ware	3025–2898 BC	n.a.	11x	U5a1b

Sample ID corresponds to the individuals described in Supplementary Material Text. Location of particular archaeological sites are shown in Fig. 1. ^*^Normal font – 14C dates, italic font- dating based on typochronology; n.a. – not analyzed.

### DNA extraction, genomic DNA library preparation and Illumina sequencing

Ancient DNA was extracted from teeth or petrous bones in a laboratory dedicated to aDNA analyses at the Department of Human Evolutionary Biology, Adam Mickiewicz University in Poznan (AMU), Poland. Molecular methods used for aDNA extraction and construction of Illumina sequencing genome libraries have been previously described^25^. One extraction and one PCR blank each were set up as negative controls during amplification of the DNA libraries. The libraries were sequenced on Illumina HiSeq2500 (125 bp, paired-end, each library on 1/10^th^ of a lane) or Illumina HiSeq X Ten (150 bp, paired-end, each library on 1/20^th^ of a lane) at the SNP & SEQ technology platform in Uppsala, Sweden.

### Mitochondrial DNA capture enrichment and Ion Torrent PGM sequencing

The RNA bait library for complete mitochondrial genomes (mtDNA) enrichment was prepared from two present day individuals of known haplotypes following^26^ with minor modifications (see Supplementary Information Text for details). Two rounds of mtDNA enrichment were carried out on 12 libraries that yielded low levels of endogenous mtDNA reads in the initial Illumina shotgun screening (Supplementary Table S1). Enriched mtDNA Illumina libraries were converted into Ion Torrent sequencing libraries by PCR with indexed fusion primers (Supplementary Table S6). PCR-amplified Ion Torrent libraries were purified, prepared into equimolar pools of up to five libraries per pool and sequenced on the Ion 318 chip using the Ion Torrent PGM system (Thermo Fisher Scientific) at Molecular Biology Techniques Laboratory, Faculty of Biology, AMU.

### Bioinformatics

Preliminary pipeline computation was undertaken using resources provided by the Swedish National Infrastructure for Computing *(*SNIC) through the Uppsala Multidisciplinary Center for Advanced Computational Science (UPPMAX)^27^. Illumina sequencing data were processed using a custom analytical pipeline^28^.

First, we trimmed residual removed adapters and merged read pairs according to^29^. BWA software package version 0.7.8^30^ was used to map merged reads as single-end reads against the revised Cambridge Reference Sequence (rCRS)^31,32^ (GenBank: NC_012920). The ratio of reads mapping to Y and X chromosomes (R_y_) (with mapping quality greater than 30) was calculated to assign molecular sex to the individuals sequenced on the Illumina platform^33^.

FASTX-Toolkit (http://hannonlab.cshl.edu/fastx_toolkit/) was used to demultiplex sequences generated by the PGM Ion Torrent. Cutadapt v.1.8.1^34^ was then used to remove long (−M 110), short (−m 35), and low-quality sequences (−q 20). The filtered reads were analyzed with FastQC v 0.11.3^35^ using the options described previously^36^. The sequences were mapped against the rCRS using TMAP v3.4.1^37^. To collapse duplicate sequence reads with identical start and end coordinates (for both PGM and Illumina sequence data) we used FilterUniqueSAMCons.py^38^. Misincorporation patterns were assessed using mapDamage v2.0.5^39^. For each individual, contamination levels were estimated with the use of schmutzi^40^ as previously in^36^. Consensus sequences were built using ANGSD v0.910^41^. We accepted only reads with a minimum mapping score of 30, a minimum base quality of 20, and a minimum coverage of 3, as in^36^. Where necessary, comparative published mt genomes were reconstructed from the bam files with the use of the same methods as described above. Mitochondrial haplogroups (mtDNA hgs) were assigned for each individual with the use of HAPLOFIND^42^, the PhyloTree phylogenetic tree build 17^43^ and Mitomaster^44^.

### Statistical analysis

For comparative studies we used ancient mtDNA data obtained from the literature, the European Nucleotide Archive (www.ebi.ac.uk/ena) and NCBI GenBank (www.ncbi.nlm.nih.gov) web databases. All comparative populations used for principal component analysis (PCA), t-Distributed Stochastic Neighbor Embedding (t-SNE), pairwise genetic distances (*F*_ST_), and AMOVA analysis are described in detail in Supplementary Tables S2–S5. We divided the population associated with the Corded Ware culture into a western group encompassing comparative German samples, and an eastern group comprising individuals linked with the Corded Ware culture from this study. Similarly individuals associated with the Yamnaya horizon were divided into western and eastern groups according to their geographic localization. Due to overlapping dating of the samples and their common origin, the western Yamnaya horizon group encompassed individuals associated with Yamnaya culture and Late Eneolithic from this study, and additional comparative samples from present day Ukraine and Bulgaria^24,45,46^. Because of potential maternal kinship between two Catacomb culture-associated individuals from this study, only one of them was used in the PCA and in a comparative Catacomb group. The eastern Yamnaya horizon group consisted of Yamnaya samples from the Samara region in Russia^14,17,46^. The map with archaeological sites from which the studied individuals originated, was generated using QGIS 2.12.2^47^ (Fig. 1).

PCA for frequencies of mtDNA hgs was calculated using Python 3.5 and Scikit-learn v. 0.18.1 package^48^. We utilized Matplotlib 1.5.1 Python package^49^ for plotting the PCA results and mtDNA hgs loadings.

We have used a centroid-based clustering approach to examine the PCA results and search for logical clusters within our data. We applied the k-means method (as implemented in Scikit-learn v. 0.18.1 Python package)^48^ to the first 5 principal components from the PCA analysis (for details see Supplementary Information Text). All k-means variants can be found in the Supplementary Material Text and Supplementary Fig. S27.

To further explore the relatedness of populations using the mtDNA hg frequencies, we ran the t-Distributed Stochastic Neighbor Embedding (t-SNE) analysis^50^ as implemented in Scikit-learn (18.1) Python package.

*F*_ST_ values were computed in Arlequin 3.5^51^. In total, 18 populations were used in *F*_ST_ and following AMOVA analyses, consisting only of ancient individuals with complete mtDNA genomes (Supplementary Table S3), using Nei’s average number of pairwise differences^52^ and 10,000 permutations to estimate the p-values. To visualize *F*_ST_ values we employed multidimensional scaling (MDS) analysis with the use of Python Scikit-learn 0.18.1 package^48^.

We have used the traditional method of analysis of molecular variance (AMOVA)^53^ to assess the population differentation using complete mt genomes. We ran AMOVA using the Arlequin 3.5 software package (for details see Supplementary Information Text).

### Availability of data and material

Mitochondrial genome sequences were deposited in GenBank under accession numbers MH176332, MH176333, MH17635 - MH176355.

## Results

### Ancient mitochondrial genomes

Out of the 45 analyzed samples, we successfully obtained 23 mtDNA genomes, belonging to individuals associated with the Corded Ware culture (N = 11), and with the Late Eneolithic (N = 3) and Bronze Age (N = 9) from western Pontic region (Table 1 and Supplementary Table S1). Eleven of the mtDNA genomes were retrieved from the Illumina shotgun screening data with the depth-of-coverage (DoC) ranging between ca. 5.4× to 64×. The remaining twelve mtDNA genomes were retrieved from the hybridization capture enrichment followed by PGM Ion Torrent sequencing, and yielded DoC ranging between 11× to 194×. Nucleotide misincorporation patterns assessed using MapDamage showed characteristic aDNA damage involving C-T and G-A transitions at the 5′ and 3′ ends of DNA fragments, respectively (Supplementary Fig. S26). Schmutzi estimations conducted for each individual showed low levels of contaminations (1–3%) (Supplementary Table S1). Additionally, we found no contamination in the extraction blanks and PCR negative controls. The mitochondrial DNA data are deposited in GenBank under accession numbers MH176332, MH176333, MH17635-MH176355.

In general, the individuals associated with the Corded Ware culture and the Yamnaya horizon were assigned to mtDNA lineages common among modern-day west Eurasian groups (hgs H, I, J, T, U2, U4, U5, W, X). Individuals associated with the Eneolithic and Yamnaya cultures were assigned to hgs U2e1a1, U5a2b, H2a1 and U5a1i1, U4c1, W3a1, W3a1a, respectively (Table 1 and Supplementary Table S1). Other Bronze Age individuals from the western Pontic region belonged to hg X4 (two individuals associated with Catacomb culture) and J2b1a, J1c2m, H1e (three individuals associated with Babyno culture). Individuals associated with the Corded Ware culture were assigned to hgs H (H6a, H15a1, H2a2, H1e), U4 (U4b1a1a, U4a2f), W5b, U5a1b, I4a and T2e (Table 1 and Supplementary Table S1).

### Genetic distances between ancient populations

**Figure 2:**
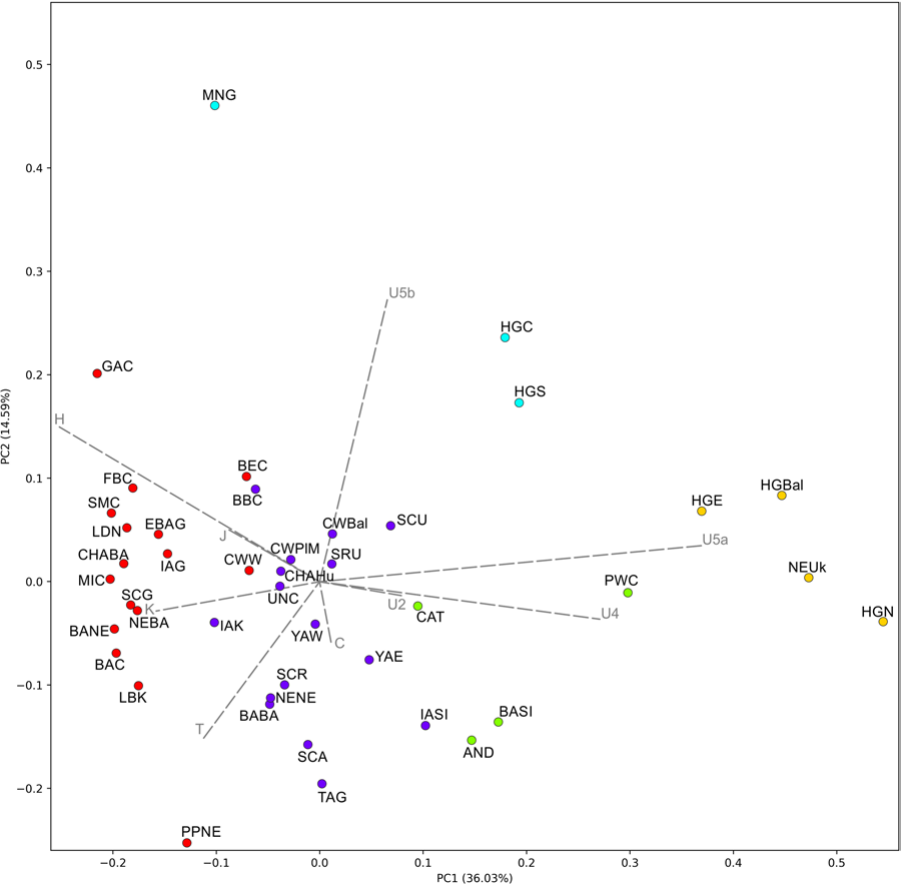
PCA based on mitochondrial DNA haplogroup frequencies with k-means clustering. The two principal components explained 50.62% of the total variance. Loading vectors, representing mitochondrial haplogroup contributions, are highlighted as grey arrows. Populations are grouped into four clusters according to k-means. Population abbreviations are as follows: BABA – Bronze Age Balkans; CAT – Catacomb Culture; CWPlM – Corded Ware Culture from Poland and Moravia; CWBal – Baltic Corded Ware Culture; IAK – Iron Age Kazakchstan; IASI – Iron Age Syberia – Aldy Bel Culture; SCA – Scytho-Siberian Pazyryk (Altai); SCR – Rostov-Scythians, Samara; SCU – Scythians from Moldova and Ukraine; TAG – Tagar Culture; GAC – Globular Amphora Culture; YAW – western Yamnaya horizon population from Ukraine and Bulgaria; YAE – eastern Yamnaya horizon population; BAC - Baalberge Culture; BANE - Bronze Age Near East; BEC - Bernburg Culture; CHAHu – Chalcolithic Hungary; CWW – Corded Ware Culture west; CHABA - Chalcolitic Balkans; EBAG - Early Bronze Age Germany; FBC – Funnel Beaker Culture; IAG – Iron Age Germany; MNG – Middle Neolithic Germany; LBK – Linear Pottery Culture; LDN – Late Danubian Neolithic; MIC - Minoans; NEBA - Neolithic Balkans; PPNE - Pre-Pottery Near East; SCG - Schöningen group; SMC - Salzmünde Culture; AND – Andronovo Culture; BASI – Bronze Age Siberia; PWC – Pitted Ware Culture; HGE – eastern hunter-gatherers; NEUk- Neolithic Ukraine; HGS – southern hunter-gatherers; HGBal – Baltic hunter-gatheres; HGC – central huther-gatherers. Detailed descriptions and references of comparative populations are provided in Supplementary Table S2.

The PCA results described 50.62% of the variability and were combined with the k-means clustering (with the *k* value of 5 as the best representation of the data, at the average silhouette of 0.2608) (Figs 2 and S27). Based on these results individuals associated with the western and eastern Yamnaya horizon (YAE and YAW in Fig. 2) were grouped within a cluster consisting of populations from central Eurasia and Europe (blue cluster) including people associated with eastern Corded Ware culture (CWPlM) and Baltic Corded Ware culture (CWBal). This cluster did not contain any populations linked with early Neolithic farmers (red), or hunter-gatherers (green and yellow). On the other hand, k-means clustering linked the western Corded Ware culture-associated population (CWW) with Near East and Neolithic farmer ancestry groups from western and central Europe.

**Figure 3:**
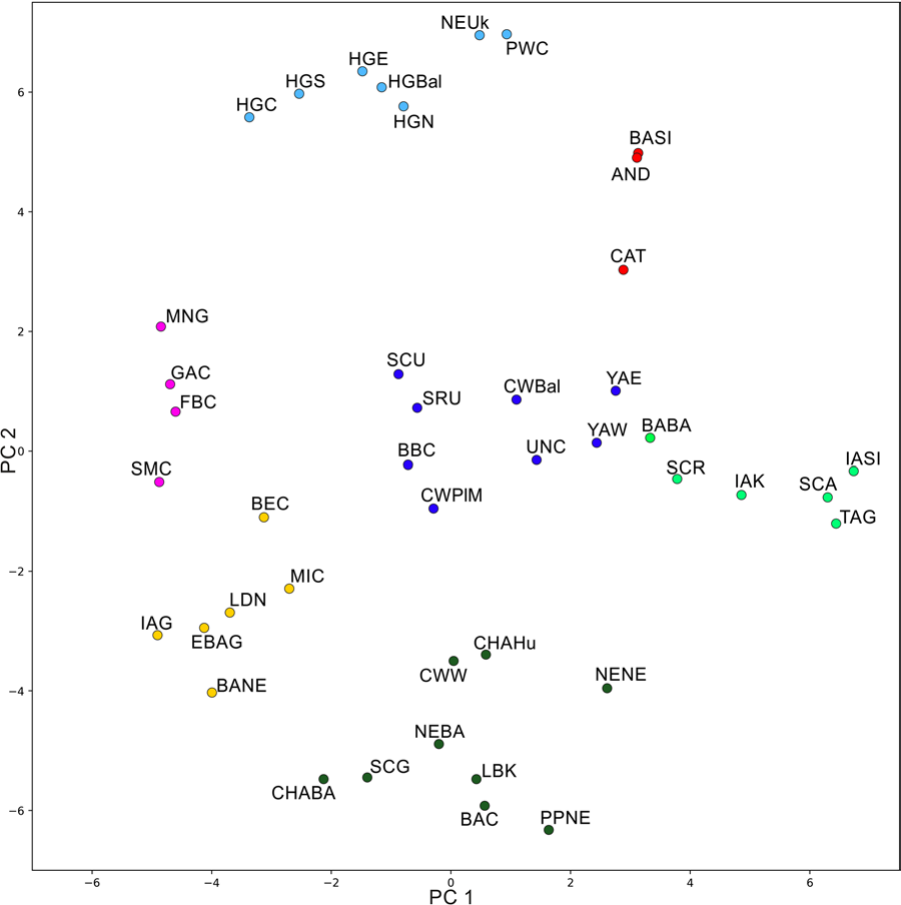
t-SNE results colored according the k-means clustering with k = 7. Population abbreviations are as in Fig. 2.

The k-means clustering on t-SNE results was consistent with the PCA results, although the approach to dimension reduction of the t-SNE algorithm is completely different than that of the PCA. Scatterplot of populations colored according to the k-means k = 7 (average silhouette 0.5158) (Fig. 3) represented the main components of European genetic ancestry. Individuals associated with western Corded Ware culture (CWW in Fig. 3) clustered with the early Neolithic Farmer ancestry group (dark green), while people associated with CWPlM from this study and CWBal showed greater affinity to the eastern European cluster (dark blue) which included mostly steppe populations associated with the Yamnaya horizon, Srubnaya, and western Scythians. Another clearly defined cluster was the central-western Asia group (red) with Andronovo, Catacomb and Siberian populations. Iron Age central Asia cluster (light green) consisted mostly of Altai and Russian Scythians and populations from Siberia and Kazakhstan. The strong hunter-gatherer ancestry cluster (light blue) included the hunter-gatherers and Neolithic populations with major hunter-gatherers component associated with the Neolithic Ukraine and the Scandinavian Pitted Ware culture. The last two clusters comprised of populations linked with the post-Linear Pottery culture from central Europe and other Middle and Late Neolithic groups from Europe (yellow and purple).

**Figure 4:**
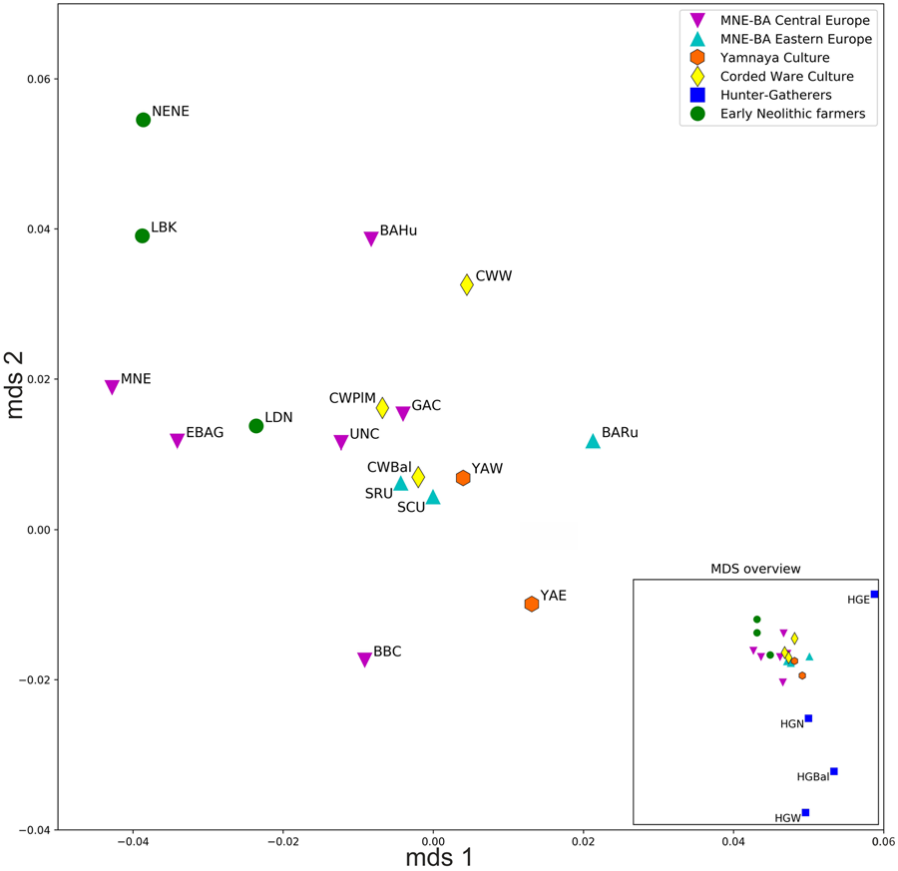
MDS plot based on *F*_ST_ values calculated from mitochondrial genomes. Population abbreviations: BBC – Bell Beaker Culture; BAHu – Bronze Age Hungary; BARu – Bronze Age Russia; CWPlM – Corded Ware Culture from Poland and Moravia; CWW – western Corded Ware Culture; CWBal – Baltic Corded Ware Culture; EBAG – Early Bronze Age Germany; GAC – Globular Amphora Culture; HGE – eastern hunter-gatherers; HGN – northern hunter-gatherers; HGW – western hunter-gatherers; HGBal – Baltic hunter-gatherers; LBK – Linear Pottery Culture; LDN – Late Danubian Neolithic; MNE – Middle Neolithic; NENE – Near Eastern Neolithic; SCU – Scythians from Moldova and Ukraine; SRU – Rostov-Scythians, Samara. Detailed information about each individual is provided in Supplementary Table S3.

Pairwise mtDNA-based *F*_ST_^54^ values (Supplementary Table S4), visualized on MDS using the raw non-linearized *F*_ST_ (stress value = 0.099) (Fig. 4), also supported the PCA results and indicated that western and eastern Yamnaya horizon groups (YAW and YAE) were closer to people associated with the eastern Corded Ware culture (CWPlM) (*F*_ST_ = 0.00; *F*_ST_ = 0.01, respectively; both p > 0.05) and Baltic Corded Ware culture (CWBal) (*F*_ST_ = 0.00; *F*_ST_ = 0.00, respectively; both p > 0.05), than to populations associated with the western Corded Ware culture (CWW) (*F*_ST_ = 0.047 and *F*_ST_ = 0.059, respectively; both statistically significant p < 0.05). Western and eastern Yamnaya horizon groups also showed close genetic affinity to the Iron Age western Scythians (SCU) (*F*_ST_ = 0.0022 and *F*_ST_ = 0.006, respectively, both p > 0.05). The most distant populations to the Yamnaya horizon groups were western hunter-gatherers (HGW) (*F*_ST_ = 0.23 and *F*_ST_ = 0.15, p < 0.001; see Supplementary Table S4).

The *F*_ST_-based MDS reflected the general European population history in the post-LGM period as the three highest *F*_ST_ scores were detected between western hunter-gatherers (HGW) and people associated with Linear Pottery culture (LBK) (*F*_ST_ = 0.33, p < 0.001), between eastern hunter-gatherers (HGE) and Baltic hunter-gatherers (HGBal) (*F*_ST_ = 0.35, p < 0.05), and between western (HGW) and eastern hunter-gatherers (HGE) (*F*_ST_ = 0.36, p < 0.05) (Fig. 4 and Supplementary Table S4). The Yamnaya horizon groups (YAE and YAW) were placed centrally between northern hunter-gatherers (HGN) and Neolithic farmers (LDN), in direct proximity to the Bronze and Iron Age populations from Eastern Europe (SCU, BARu, SRU) and close to individuals associated with eastern and Baltic Corded Ware culture (Fig. 4).

We investigated the within- and between-group variability using an AMOVA analysis. Concentrating on the eastern and western Corded Ware groups, we found the best variability distribution when the individuals associated with the western Corded Ware culture (CWW in Supplementary Table S5) were grouped together with the Middle Neolithic/Bronze Age Central Europe groups, while individuals associated with the eastern and Baltic Corded Ware culture (CWPlM, CWBal), and Yamnaya horizon groups (YAW and YAE) clustered together with the eastern Europe populations (from the Middle Neolithic-Bronze Age) (4.68% of variability among groups, 3.04% among populations within groups).

## Discussion

By analyzing ancient mitochondrial genomes, we show that people from the eastern and western Corded Ware culture were genetically differentiated. Individuals associated with the eastern Corded Ware culture (from present day Poland and the Czech Republic) shared close maternal genetic affinity with individuals associated with the Yamnaya horizon while the genetic differentiation between individuals associated with the western Corded Ware culture (from present-day Germany) and the Yamnaya horizon was more extensive. This decreasing cline of steppe related ancestry from east to west likely reflect the direction of the steppe migration. It also indicates that more people with steppe-related ancestry, likely both females and males, contributed to the formation of the population associated with the eastern Corded Ware culture. Similarly, closer genetic affinity to populations associated with Yamnaya horizon can be observed in Baltic Corded Ware groups, which confirms earlier indications of a direct migrations from the steppe not only to the west but also to the north, into the eastern Baltic region^18,19,55^. The mitochondrial data further suggests that with increased distance from the source populations of the steppe, the contribution of local people increase, which is seen as an increase of maternal lineages of Neolithic farmer ancestry in individuals associated with the western Corded Ware culture.

Among the analyzed samples, we identified two Catacomb culture-associated individuals (poz220 and poz221) belonging to hg X4. They are the first ancient individuals assigned to this particular lineage. Haplogroup X4 is rare among present day populations and has been found only in one individual each from Central Europe, Balkans, Anatolia and Armenia^56,57^. Moreover, we have reported mtDNA haplotypes that might be associated with the migration from the steppe and point to genetic continuity in the north Pontic region from Bronze Age until the Iron Age. These haplotypes were assigned to hgs U5, U4, U2 and W3. MtDNA hgs U5a and U4, identified in this study among Yamnaya, Late Eneolithic and Corded Ware culture-associated individuals, have previously been found in high frequencies among northern and eastern hunter-gatherers^19,23,28,55,58,59^. Moreover, they appeared in the north Pontic region in populations associated with Mesolithic (hg U5a)^45^, Eneolithic (Post-Stog) (hg U4)^24^, Yamnaya (hgs U5, U5a)^24^, Catacomb (hgs U5 and U5a)^24^ and Iron Age Scythians (hg U5a)^60^, suggesting genetic continuity of these particular mtDNA lineages in the Pontic region from, at least, the Bronze Age. Hgs U5a and U4-carrying populations were also present in the eastern steppe, along with individuals from the Yamnaya culture from Samara region^14,17^, the Srubnaya^23^ and the Andronovo from Russia^14^. Interestingly, hg U4c1 found in the Yamnaya individual (poz224) has so-far been found only in two Bell Beaker- associated individuals^61^ and one Late Bronze Age individual from Armenia^14^, which might suggest a steppe origin for hg U4c1. A steppe origin can possibly also be assigned to hg U4a2f, found in one individual (poz282) but not reported in any other ancient populations to date, and to U5a1- the ancestral lineage of U5a1b, reported for individual poz232, which was identified not only in Corded Ware culture-associated population from central and eastern Europe^55,61^ but also in representatives of Catacomb culture from the north Pontic region^24^, Yamnaya from Bulgaria and Russia^17,46^, Srubnaya^23^ and Andronovo^62^-associated groups. Hg U2e, reported for Late Eneolithic individual (poz090), was also identified in western Corded Ware culture-associated individual^23^ and in succeeding Sintashta^14^, Potapovka and Andronovo^23^ groups, suggesting possible genetic continuity of U2e1 in the western part of the north Pontic region.

Hgs W3a1 and W3a1a, found in two Yamnaya individuals from this study (poz208 and poz222), were also identified in Yamnaya-associated individuals from the Russia Samara region^17^ and later in Únětice and Bell Beaker groups from Germany^61,63^, supporting the idea of an eastern European steppe origin of these haplotypes and their contribution to the Yamnaya migration toward the central Europe. The W3a1 lineage was not identified in Neolithic times and, thus, we assume that it appeared in the steppe region for the first time during the Bronze Age. Notably, hgs W1 and W5, which predate the Bronze Age in Europe, were found only in individuals associated with the early Neolithic farmers from Starčevo in Hungary (hg W5)^64^, early Neolithic farmers from Anatolia (hg W1-T119C)^23^, and from the Schöningen group (hg W1c)^61^ and Globular Amphora culture from Poland (hg W5)^45^.

This study is the first to present mitochondrial genome data from the population associated with Corded Ware culture from the south-eastern part of present-day Poland. As this area is geographically close to the steppe region, it provides us with a better picture of the early steppe migration between 3,000 and 2,500 BC. Although our results indicate a contribution of females as well as males to the formation of populations associated with eastern Corded Ware culture, more detailed studies of X chromosome data are needed to clearly resolve female and male migrations, especially between the western Pontic steppe and the eastern part of the North European Plain.

## Conclusions

Ancient mitochondrial genome data from the western Pontic region and, for the first time, from the south-eastern part of present day Poland, show close genetic affinities between populations associated with the eastern Corded Ware culture and the Yamnaya horizon. This indicates that females had also participated in the migration from the steppe. Furthermore, greater mtDNA differentiation between populations associated with the western Corded Ware culture and the Yamnaya horizon points to an increased contribution of individuals with a maternal Neolithic farmer ancestry with increasing geographic distance from the steppe region, forming the population associated with the western Corded Ware culture. Among the analyzed samples, we identified, for the first time in ancient populations, two Catacomb culture-associated individuals belonging to the now-rare mtDNA hg X4.

## Electronic supplementary material

Supplementary Tables S1-S5

Supplementary Information Text

## References

[1] Ivanova SV (2015). ‘Yampil Inspirations’: A Study of the Dniester Cultural Contact Area at the Frontier of Pontic and Baltic Drainage Basins. Balt.-Pontic Stud..

[2] Włodarczak, P. Sekwencja czynności obrzędowych: problem korespondencji tradycji funeralnych kultury jamowej i kultury ceramiki sznurowej na Wyżynie Podolskiej. In *Naddniestrzańskie kompleksy cmentarzysk kurhanowych społeczności z III i z pierwszej połowy II tysiąclecia przed Chr. w okolicach Jampola, obwód winnicki. Z badań nad północno-zachodnią rubieżą osadnictwa społeczności kręgu kultur “wczesnobrązowych” strefy pontyjskiej. Badania z lat 1984–2014*. 314–340 (Archeologia Bimaris - Monografie 6, 2014).

[3] Ivanova SV, Toschev GNL (2015). Eneolithic and Bronze Age prologue Pontic societies. Forest-steppe middle Dniester and Prut drainage basins in the 4th/3rd-2nd millenium BC: a history of investigations. Balt.-Pontic Stud..

[4] Goslar, T., Klochko, V. I., Kośko, A., Włodarczak, P. & Żurkiewicz, D. Chronometry of Late Eneolithic and ‘Early Bronze’ Cultures in the Middle Dniester Area: Investigations of the Yampil Barrow Complex. In *Baltic-Pontic Studies* **20**, 257–292 (2015).

[5] Kośko A, Klochko VI (2009). Transit routes between the Baltic and Black Seas: early development stages – from the 3rd to the middle of the 1st millenium BC. An outline of research project. Balt.-Pontic Stud..

[6] Morgunova N, Khokhlova O (2013). Chronology and Periodization of the Pit-Grave Culture in the Area Between the Volga and Ural Rivers Based on 14C Dating and Paleopedological Research. Radiocarbon.

[7] Kośko, A. Influences of the ‘Pre-Yamnaya’ (‘Pre-Pitgrave’) Communities from the Black Sea Steppe Area in Western European Cultures. *Lénéolithique Début Lâge Bronze Dans Certain. Régions Eur* (1985).

[8] Rassamakin, Y. Y. The main directions of the development of early pastoral societies of northern Pontic Zone: 4500–2450 BC (Pre-Yamnaya Cultures and Yamnaya Culture). In *Baltic-Pontic Studies* **2**, 29–70 (1994).

[9] Rassamakin, Y. Y. The Eneolithic of the Black Sea steppe: dynamics of cultural and economic development 4500–2300 BC. In *Late Prehistoric exploitation of the Eurasian steppe* 59–182 (Cambridge, 1999).

[10] Shaposhnikova, O. G. Yamnaya kulturno-istoricheskaya obchnost. *Arkheologiya Ukr. SSR* 336–352 (1985).

[11] Rassamakin, Y. Y. & Nikolova, A. Carpathian Imports and Imitations in Context of the Eneolithic and Early Bronze Age of the Black Sea Steppe Are. *Import Imitation Archaeol*. 51–87 (2008).

[12] Anthony, D. W. *The Horse, the Wheel, and Language: How Bronze-Age Riders from the Eurasian Steppes the Modern World*. (Princeton, Oxford: Princeton University Press, 2007).

[13] Kośko, A. Eastern European Context for Studies on the Use of Wagons in the Baltic Sea Catchment Area of the 4th and 3th Millennia BC. *Environ. Subsist. - Forty Years Janusz Kruks Settl. Stud*. 429–440 (2013).

[14] Allentoft ME (2015). Population genomics of Bronze Age Eurasia. Nature.

[15] Kośko A, Klochko VI (2013). The Baltic Drainage Basin in the Recenstruction of the Map of Central Europe Held in Common by Northern - Pontic Early - Bronze Civilization Communities; 3200–1600 BC. An Outline of the Research Programme. Balt.-Pontic Stud..

[16] Gimbutas, M. The First Wave of Eurasian Steppe Pastoralists into Copper Age Europe. *J. Indo-Eur. Stud*. 277–337 (1977).

[17] Haak W (2015). Massive migration from the steppe was a source for Indo-European languages in Europe. Nature.

[18] Mittnik A (2018). The genetic prehistory of the Baltic Sea region. Nat. Commun..

[19] Saag L (2017). Extensive Farming in Estonia Started through a Sex-Biased Migration from the Steppe. Curr. Biol..

[20] Lazaridis I (2016). Genomic insights into the origin of farming in the ancient Near East. Nature.

[21] Goldberg, A., Günther, T., Rosenberg, N. A. & Jakobsson, M. Ancient X chromosomes reveal contrasting sex bias in Neolithic and Bronze Age Eurasian migrations. *Proc. Natl. Acad. Sci*. **114**, 2657–2662 (2017). 10.1073/pnas.1616392114 PMC5347611 28223527

[22] Kristiansen K (2017). Re-theorising mobility and the formation of culture and language among the Corded Ware Culture in Europe. Antiquity.

[23] Mathieson I (2015). Genome-wide patterns of selection in 230 ancient Eurasians. Nature.

[24] Nikitin AG, Ivanova S, Kiosak D, Badgerow J, Pashnick J (2017). Subdivisions of haplogroups U and C encompass mitochondrial DNA lineages of Eneolithic–Early Bronze Age Kurgan populations of western North Pontic steppe. J. Hum. Genet..

[25] Juras A (2017). Investigating kinship of Neolithic post-LBK human remains from Krusza Zamkowa, Poland using ancient DNA. Forensic Sci. Int. Genet..

[26] Carpenter ML (2013). Pulling out the 1%: Whole-Genome Capture for the Targeted Enrichment of Ancient DNA Sequencing Libraries. Am. J. Hum. Genet..

[27] Lampa S, Dahlö M, Olason PI, Hagberg J, Spjuth O (2013). Lessons learned from implementing a national infrastructure in Sweden for storage and analysis of next-generation sequencing data. Giga Science.

[28] Günther T (2015). Ancient genomes link early farmers from Atapuerca in Spain to modern-day Basques. Proc. Natl. Acad. Sci..

[29] Meyer M, Kircher M (2010). Illumina sequencing library preparation for highly multiplexed target capture and sequencing. Cold Spring Harb. Protoc..

[30] Li H, Durbin R (2009). Fast and accurate short read alignment with Burrows-Wheeler transform. Bioinforma. Oxf. Engl..

[31] Anderson S (1981). Sequence and organization of the human mitochondrial genome. Nature.

[32] Andrews RM (1999). Reanalysis and revision of the Cambridge reference sequence for human mitochondrial DNA. Nat. Genet..

[33] Skoglund P, Storå J, Götherström A, Jakobsson M (2013). Accurate sex identification of ancient human remains using DNA shotgun sequencing. J. Archaeol. Sci..

[34] Martin M (2011). Cutadapt removes adapter sequences from high-throughput sequencing reads. EMBnet.journal.

[35] Andrews, S. A quality control tool for high throughput sequence data (2012).

[36] Chyleński M (2017). Late Danubian mitochondrial genomes shed light into the Neolithisation of Central Europe in the 5th millennium BC. BMC Evol. Biol..

[37] Merriman B, Ion Torrent R, Team D, Rothberg JM (2012). Progress in ion torrent semiconductor chip based sequencing. Electrophoresis.

[38] Kircher, M. Analysis of High-Throughput Ancient DNA Sequencing Data. In Ancient DNA SE - 23 Methods in Molecular Biology. In *Ancient DNA: Methods and Protocols* (Humana Press, 2012). 10.1007/978-1-61779-516-9_23 22237537

[39] Jónsson H, Ginolhac A, Schubert M, Johnson PLF, Orlando L (2013). mapDamage2.0: fast approximate Bayesian estimates of ancient DNA damage parameters. Bioinforma. Oxf. Engl..

[40] Renaud G, Slon V, Duggan AT, Kelso J (2015). Schmutzi: estimation of contamination and endogenous mitochondrial consensus calling for ancient DNA. Genome Biol..

[41] Korneliussen TS, Albrechtsen A, Nielsen R (2014). ANGSD: Analysis of Next Generation Sequencing Data. BMC Bioinformatics.

[42] Vianello D (2013). HAPLOFIND: a new method for high-throughput mtDNA haplogroup assignment. Hum. Mutat..

[43] van Oven M, Kayser M (2009). Updated comprehensive phylogenetic tree of global human mitochondrial DNA variation. Hum. Mutat..

[44] Lott MT (2013). mtDNA Variation and Analysis Using Mitomap and Mitomaster. Curr. Protoc. Bioinforma..

[45] Mathieson, I. *et al*. The genomic history of southeastern Europe. *Nature* (2018). 10.1038/nature25778 PMC6091220 29466330

[46] Wilde S (2014). Direct evidence for positive selection of skin, hair, and eye pigmentation in Europeans during the last 5,000 y. Proc. Natl. Acad. Sci. USA.

[47] QGIS Development Team. *QGIS Geographic Information System. Open Source Geospatial Foundation Project* (2015).

[48] Pedregosa F (2011). Scikit-learn: Machine Learning in Python. J. Mach. Learn. Res..

[49] Hunter JD (2007). Matplotlib: A 2D Graphics Environment. Comput. Sci. Eng..

[50] van der Maaten L, Hinton G (2008). Visualizing Data using t-SNE. J. Mach. Learn. Res..

[51] Excoffier L, Lischer HEL (2010). Arlequin suite ver 3.5: a new series of programs to perform population genetics analyses under Linux and Windows. Mol. Ecol. Resour..

[52] Nei M, Li WH (1979). Mathematical model for studying genetic variation in terms of restriction endonucleases. Proc. Natl. Acad. Sci. USA.

[53] Excoffier L, Smouse PE, Quattro JM (1992). Analysis of molecular variance inferred from metric distances among DNA haplotypes: application to human mitochondrial DNA restriction data. Genetics.

[54] Slatkin M (1995). A measure of population subdivision based on microsatellite allele frequencies. Genetics.

[55] Jones ER (2017). The Neolithic Transition in the Baltic Was Not Driven by Admixture with Early European Farmers. Curr. Biol. CB.

[56] Röck AW, Dür A, van Oven M, Parson W (2013). Concept for estimating mitochondrial DNA haplogroups using a maximum likelihood approach (EMMA). Forensic Sci. Int. Genet..

[57] Fernandes V (2012). The Arabian Cradle: Mitochondrial Relicts of the First Steps along the Southern Route out of Africa. Am. J. Hum. Genet..

[58] Skoglund P (2014). Genomic diversity and admixture differs for Stone-Age Scandinavian foragers and farmers. Science.

[59] Skoglund P (2012). Origins and genetic legacy of Neolithic farmers and hunter-gatherers in Europe. Science.

[60] Juras, A. *et al*. Diverse origin of mitochondrial lineages in Iron Age Black Sea Scythians. *Sci. Rep*. **7**, 43950 (2017). 10.1038/srep43950 PMC5339713 28266657

[61] Brandt G (2013). Ancient DNA reveals key stages in the formation of central European mitochondrial genetic diversity. Science.

[62] Keyser C (2009). Ancient DNA provides new insights into the history of south Siberian Kurgan people. Hum. Genet..

[63] Olalde I (2018). The Beaker phenomenon and the genomic transformation of northwest Europe. Nature.

[64] Lipson M (2017). Parallel palaeogenomic transects reveal complex genetic history of early European farmers. Nature.

