# Supplementary material for: Mitochondrial genomes reveal an east to west cline of steppe ancestry in Corded Ware populations
Source: Sci Rep. 2018 Aug 2;8:11603. doi: 10.1038/s41598-018-29914-5 (PMC6072757; doi:10.1038/s41598-018-29914-5)
Supplement: Supplementary file 2 — Supplementary Information Text [file 41598_2018_29914_MOESM2_ESM.pdf]

Supplementary Information Text

Mitochondrial genomes reveal an east to west cline in steppe ancestry in corded ware populations

Anna Juras[1*], Maciej Chyleński[2], Edvard Ehler[1,3], Helena Malmström[4,15], Danuta Żurkiewicz[2], Piotr Włodarczak[5], Stanisław Wilk[6], Jaroslav Peška[7,8], Pavel Fojtík[9], Miroslav Králík[10], Jerzy Libera[11], Jolanta Bagińska[12], Krzysztof Tunia[5], Viktor I. Klochko[13], Miroslawa Dabert[14], Mattias Jakobsson[4,15], Aleksander Keśko[2]

[1] Department of Human Evolutionary Biology, Institute of Anthropology, Faculty of Biology, Adam Mickiewicz University in Poznan, Umultowska 89, 61-614 Poznań, Poland

[2]Institute of Archaeology, Faculty of History, Adam Mickiewicz University in Poznan, Umultowska 89D, 61-614 Poznań, Poland

[3]Laboratory of Genomics and Bioinformatics, Institute of Molecular Genetics of the ASCR, v. v. i., Vídeňská 1083, 142 20 Prague 4, Czech Republic

[4]Human Evolution, Department of Organismal Biology and SciLifeLab, Uppsala University, Norbyvägen 18C, SE-752 36 Uppsala, Sweden

[5]Polish Academy of Sciences, Institute of Archaeology and Ethnology, Sławkowska str. 17, 31-016 Kraków, Poland

[6]Institute of Archaeology, Jagiellonian University, Gołębia 11, 31-007 Kraków, Poland

[7] Archaeological Centre Olomouc, U Hradiska 42/6, 779 00 Olomouc, Czech Republic

[8] Department of History - Section of Archaeology, Philosophical faculty, Palacký University Olomouc, Na Hradě 5, 771 80 Olomouc, Czech Republic

[9]Institute of Archaeological Heritage Brno, v.v.i., Kaloudova 30, 614 00 Brno, Czech Republic

[10]Laboratory of Morphology and Forensic Anthropology (LaMorFA), Department of Anthropology, Faculty of Science, Masaryk University, Kotlářská 267/2, 611 37 Brno, Czech Republic

[11]Institute of Archaeology, Maria Curie-Skłodowska University, Maria Curie-Skłodowska Square 4, 20-031 Lublin, Poland

[12]Muzeum Regionalne im. Janusza Petera, ul. Zamojska 2, 22-600 Tomaszów Lubelski, Poland

[13]National University of "Kyiv-Mohyla Academy", Institute of Archaeology, Hryhoriya Skovorody St. 2, 04070 Kyiv, Ukraina

[14]Molecular Biology Techniques Laboratory, Faculty of Biology, Adam Mickiewicz University in Poznan, Umultowska 89, 61-614 Poznań, Poland

[15]Centre for Anthropological Research, University of Johannesburg, Auckland Park, 2006 Johannesburg South Africa

* Corresponding authors: Anna Juras, PhD, tel.: +48 61 829 56 25, fax: +48 61 829 57 30, e-mail: annaj@amu.edu.pl

Supplementary Information Text (Materials and Methods)

Materials and archaeological background

Yampil Barrow Complex

In the cultural landscape of the Middle Dniester, Yampil (Vinnitsa region), the concentration of barrow features can be clearly discerned, one delineating the north-west boundary of their forest-steppe radius. Initial excavation of Yampil barrows was begun in the context of conservation programs in 1984 for the purposes of identifying the role of their creators (in the decided majority) of Yamnaya culture (Pit Grave culture) communities and the context of later users from the communities of the Catacomb and Babyno cultures as well as other groups from the younger periods of prehistory.

At the beginnings of the 21st century, the context of the Kyiv-Poznań programme of studies "*pograniczem biokulturowym Wschodu and Zachodu Europu*" (The Biocultural Borderland Between the West and East of Europe) (Kośko 2009), the project "*badań początków szlaków bałtycko–pontyjskiego międzymorza: IV-I tys. BC*" (Research on the Beginnings of Baltic-Pontic Routes Between the Seas: 4th to the Ist Mill.) was started. These studies were supported by NCN grant project no 211/01/M/HS3/02142. The research aimed to undertake also an archival search of *the Boh valley region communication routes*, during which in the archaeological collections of the County Regional Lore Museum, Vinnitsa, three vessels were documented in the inventories of Yamnaya culture inhumations. These pointed to stylistic associations with so-called Turin amphorae bearing genetic origins from the central Europe Corded Ware culture circle.

This in association with their borderland location, in terms of the western reach of the Yamnaya culture, the maximum proximity to the open 'corded' settlement network of the Upper Dniester, was responsible for a systemic interest in the genetic context of source complexes here[1].

In 2010 the Yampil Excavation Expedition was established, whose task was the research of the representative series of sites. Up to 2014 seven barrows in all were examined on four archaeological sites (Supplementary Fig S1)[2].

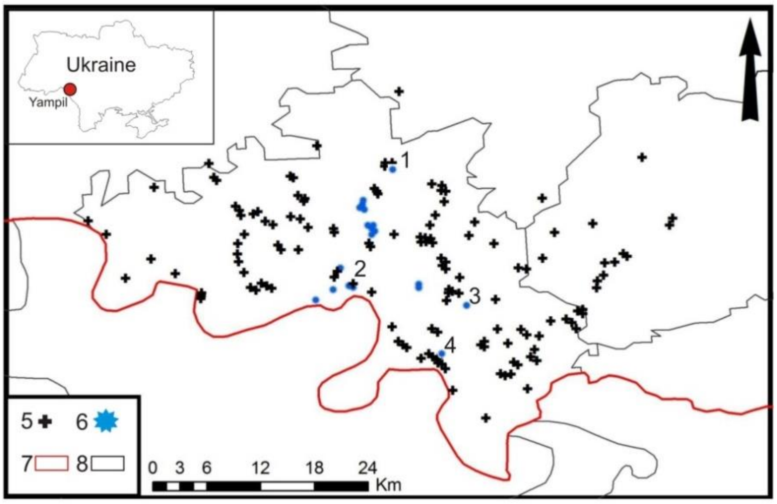

Supplementary Figure S1. Map of Yampol Barrow Complex.

Pidlisivka barrow 1, Yampil Region, Vinnitsa Oblast, Ukraine

Research on the barrow was conducted in 2010. The site is located near the village of Pidlisivka, on the high plateau on the right bank of the River Jałanka, north-west of its mouth into the River Markivki, tributary of the Dniester – some 7 km from the corridor of the latter. At the commencement of barrow examination the tumulus was not a distinct form in the landscape. In the immediate surrounds of the mound the presence of vast signs of destruction caused by trenches from World War II was observed (features 2, 3 and 14). The recorded maximum height did not exceed 1 m in relation to the surrounding landscape and the recorded diameter of the tumulus on a west-east axis was measured at 30 m approximately.

Under the mound of Barrow 13 inhumations were discovered (Supplementary Fig S2) and samples taken from five of the features for DNA analysis (1A, 1B, 5, 11, 13), which in the case of 1B and 13 DNA was obtained successfully.

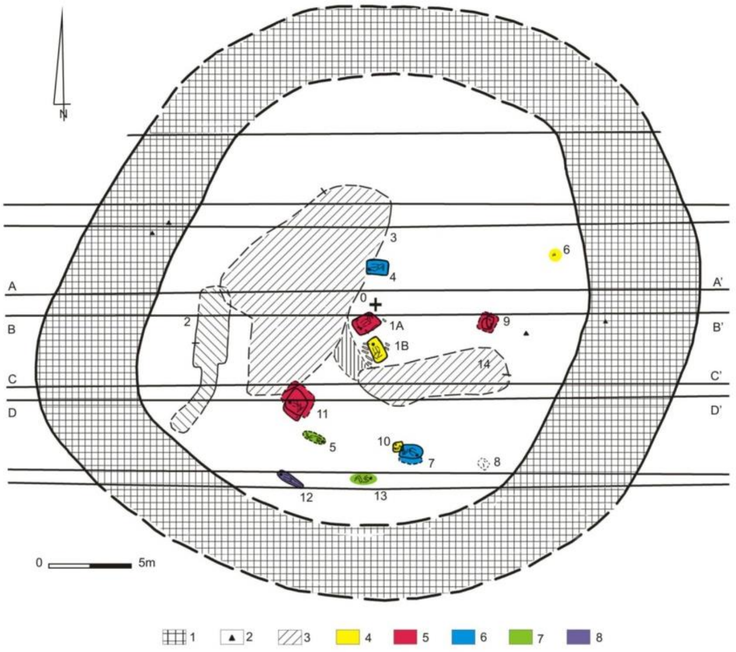

Supplementary Figure S2. Barrow 1 from Pidlisivka site. 1- barrow ditch; 2 –pottery shards; 3- present-day damage; yellow – Eneolithic features; red – Yamnaya culture features; blue – Catacomb culture features; green – Babyno Culture features; purple – Iron Age features.

Grave 1B (Eneolithic) – poz090

Feature 1B is most likely the oldest central inhumation associated with Eneolithic communities. The male, aged 22 to 25, was interred along a north-west to south-east axis in a supine position with knees raised (Supplementary Fig S3). The deceased was laid to rest on a mat made out of organic materials and the only readable elements of grave furnishings were ochre and a flint flake. Over the rectangular grave pit measuring 1.45 x 0.7 m a roof of oak planks was constructed at an angle to the longer axis of the grave.

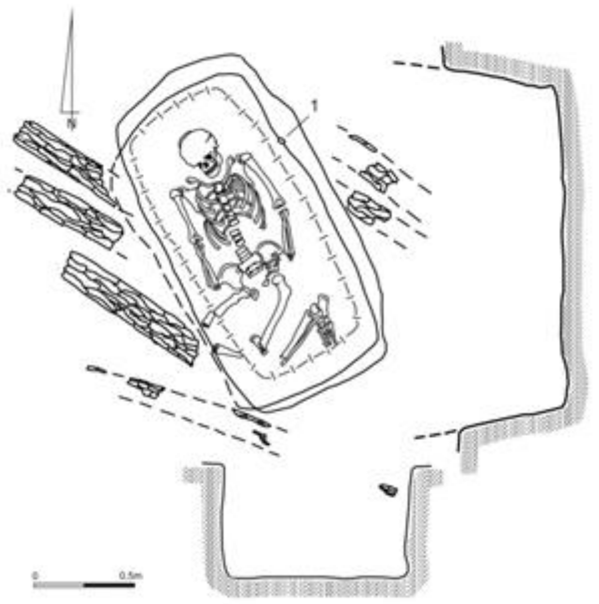

Supplementary Figure S3. Grave 1B, Pidlisivka barrow1.

Grave 13 (Babyno culture) - poz094

Feature 13 is the grave of a woman aged around 25. Excavations at the southern edge of the tumulus mound were conducted for the phase of exploitation by the communities of the Babyno culture. The deceased lay on her right side in a crouched position on an east-west axis. No additional elements of grave furnishings were recorded near the human remains. From above the grave was secured by a fragment of a chalk slab.

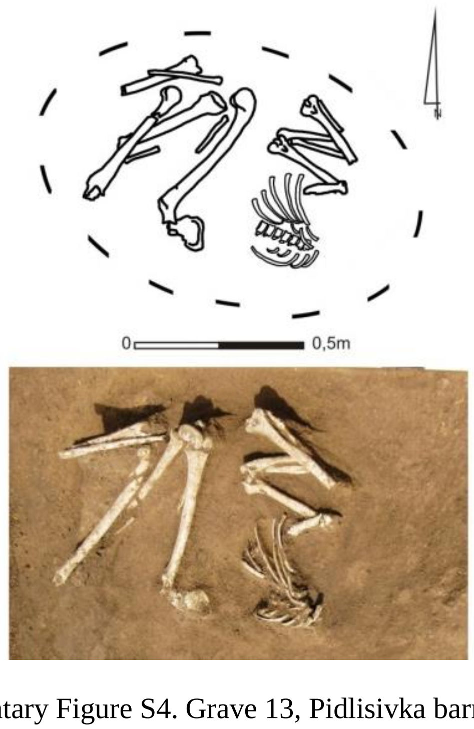

Supplementary Figure S4. Grave 13, Pidlisivka barrow 1.

Porohy 3A, Yampil Region, Vinnitsa Oblast, Ukraine

Barrow 3A in Porohy is situated in a concentration of 5 tumuli in the area of the Dniester watershed and Rusava River at a distance of 1.5 km from the former. At the time of commencing excavation work the actual form of the mound was already significantly eroded, its height amounting to approximately 1.2 m and its diameter approximately 40 m. Archaeological investigation at this site was conducted in 2011 and as a consequence 22 features associated with the Eneolithic were documented (4, 6, 7, 9, 13 14, 18), YC (1, 2, 10, 11, 12, 15, 16, 17, 19, 20), NC (3, 5, 8, 22) and that of the Iron Age - feature 21. For the purposes of DNA analysis samples were taken from the following features: 1, 3, 5, 7, 10, 11, 12, 14, 15, 19, 20 and 22. Only in the case of feature 19 DNA was obtained successfully.

Grave 19 (Yamnaya culture) – poz208

This is the grave of a deceased child no older than one and a half years (Supplementary Figure S5). The individual lay supine along a west-east axis. The grave pit, measuring 0.9×0.4 m, was most likely secured by a wooden lid. Under the bones in the grave the remains of an organic mat were discovered as well as traces of ochre on the skull.

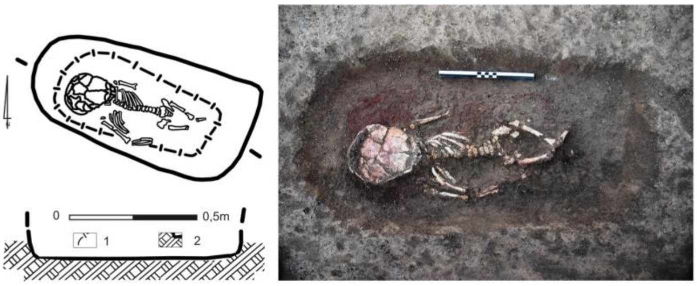

Supplementary Figure S5. Grave 19, Porohy barrow 3A.

Klembivka barrow 1, Yampil Region, Vinnitsa Oblast, Ukraine

Site 1 in Klembivka is located on a high watershed bound by the valleys of the Rusava and Korytna rivers**.** Of particular note is its clear separation from the valley of the Dniester (about 15 km) in relation to the remaining sites under discussion here. This tumulus was part of a concentration of five other mounds. At the time of commencing excavation work in 2012 the height of the mound amounted to approximately 0.7 m and that of the diameter about 24 m.

The oldest central inhumation (grave 14) and two further graves (no. 5 and 15) were associated with the Neolithic creators of this settlement (Supplementary Fig 6). Subsequent settlers of the area used for cult purposes are associated with the communities of the Babyno culture. The central inhumation for this stage is feature 1 and other associate inhumations: 2, 3, 6, 8, 10. The chronological sequence here is completed by the communities of the Noua culture for whom the major site was feature 13, while the remaining graves (features 7, 11, 12) were located at a significant distance away from the initial site, within the barrow ditch.

DNA was successfully obtained for all the samples taken for this purpose, forming a picture for each of the stages presented above (features 5, 14, 3, 11, 12).

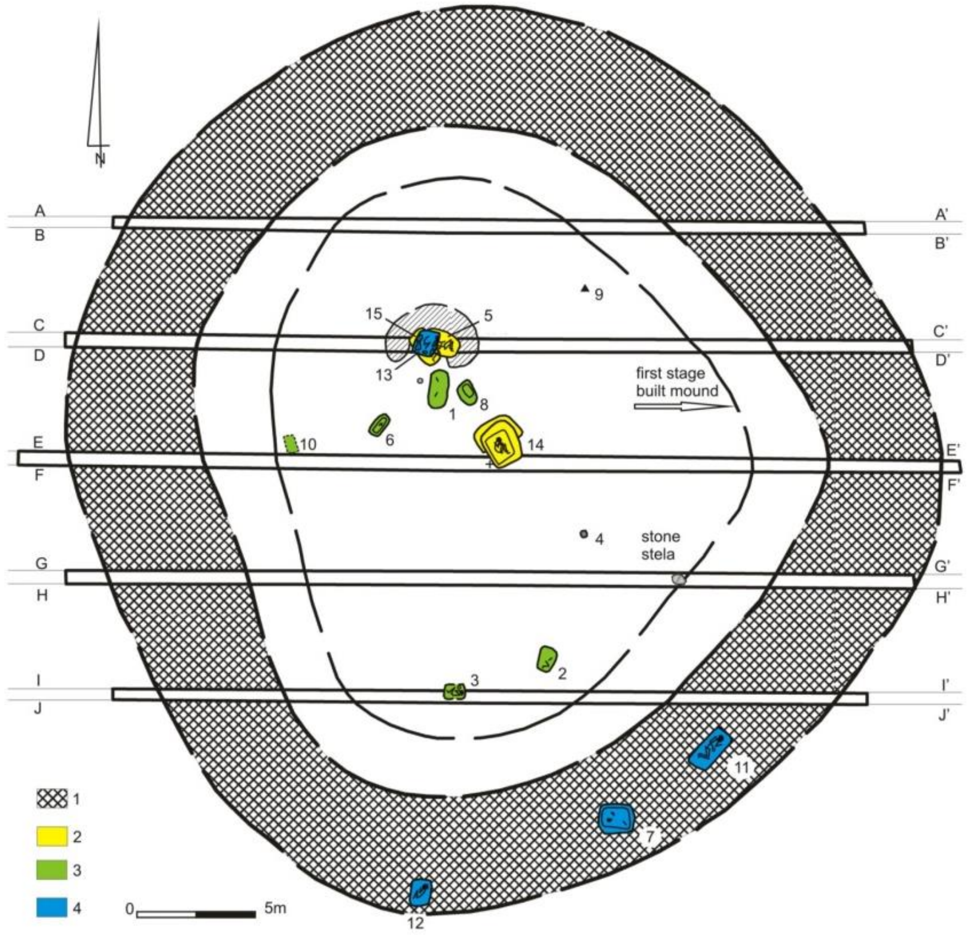

Supplementary Figure S6. Barrow 1 from Klembivka site. 1- barrow ditch; yellow – Eneolithic features; green – Babyno culture features; blue – Noua culture features.

Grave 3 (Babyno culture) – poz356

A deceased male aged 35-45 lay at the bottom of the pit, measuring 0.9 times a .6 m along an east-west axis (Supplementary Fig S7). The skeleton was poorly preserved and in a crouched position on its right side. The only element of furnishing was a small ceramic vessel deposited at the bones of the upper limbs.

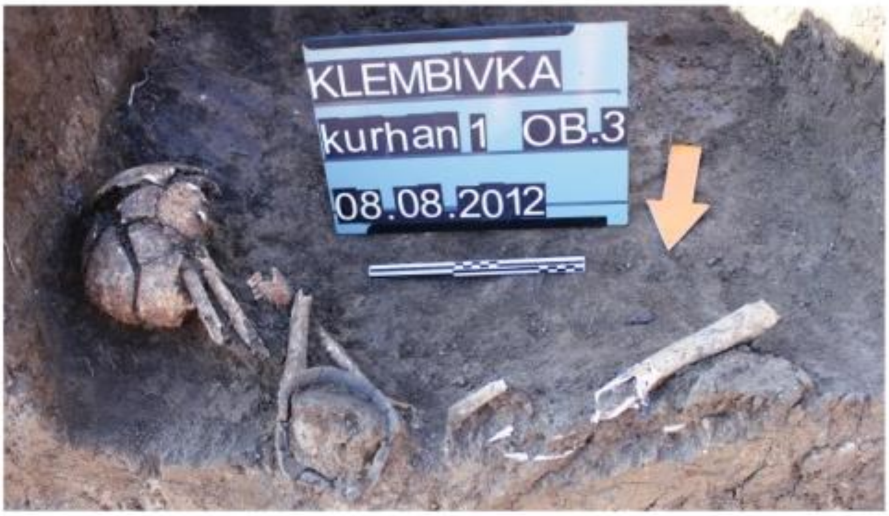

Supplementary Figure S7. Grave 3, Klembivka barrow 1.

Grave 5 (Eneolithic) – poz211

In a pit measuring 1.85×0.95 m, along a west-east axis, there lay a well preserved male skeleton aged 50-55, supine, crouched and head and lower limbs turned towards the left (Supplementary Fig S8). The grave did not show any signs of furnishings and neither were there any wooden parts of construction for the roofing of the pit.

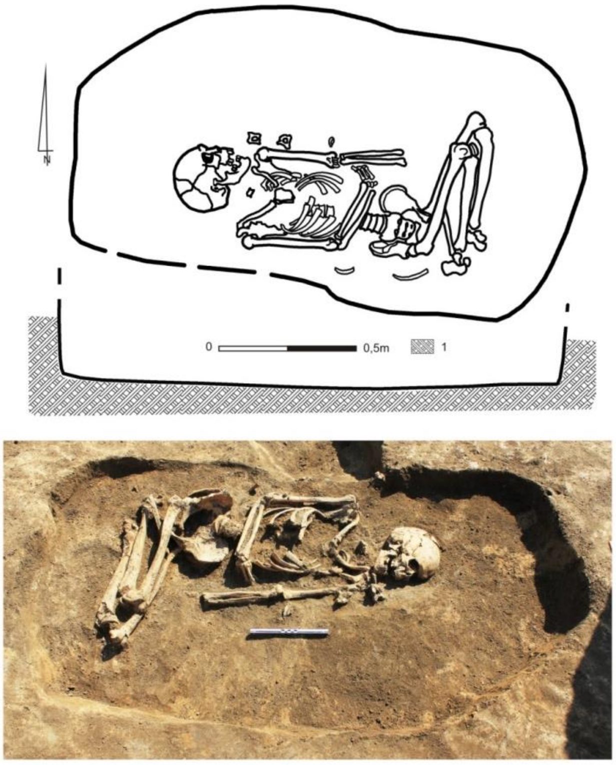

Supplementary Figure S8. Grave 5, Klembivka barrow 1.

Grave 12 (Babyno culture) – poz213

The skeleton of a deceased child, aged 12 to 14, lay in a crouched position at the bottom of the pit, which measured 1.0×0.8 m along a north-south axis (Supplementary Fig S9). From above the grave was secured by a layer of limestone.

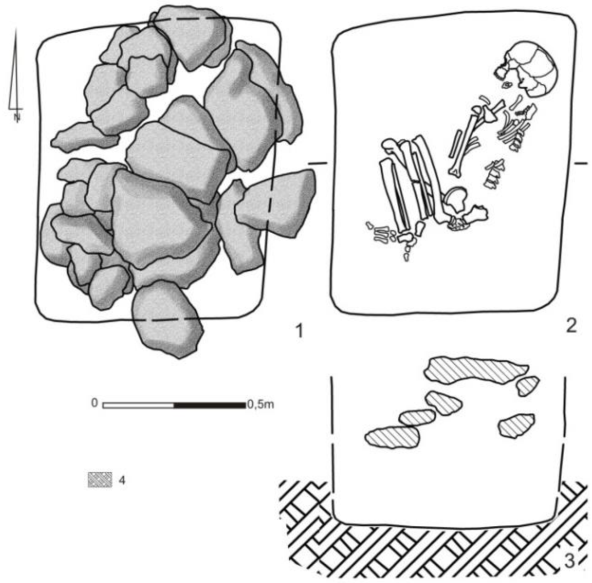

Supplementary Figure S9. Grave 12, Klembivka barrow 1.

Grave 14 (Eneolithic- central grave) – poz214

At the bottom of a deep pit, measuring 2.05×1.95 m, there lay a well preserved skeleton of a male aged 25-30 with signs of red ochre (Supplementary Figure S10). The deceased was placed in a crouched position on his left side and most likely rested on a mat of organic materials. On the occipital bone and the right parietal an irregular opening was present, the result of a blow (most likely the cause of death). Between the bones of the ribs a small flint flake was found. The pit could have been furnished with additional elements of construction made out of wood.

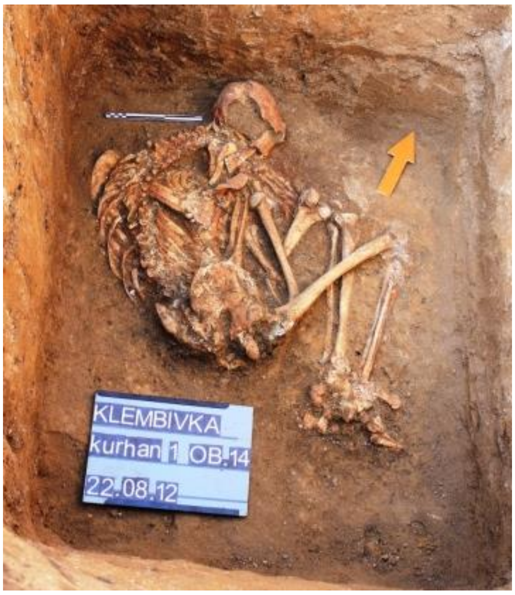

Supplementary Figure S10. Grave 14, Klembivka barrow 1.

Prydnistryanske, site 1, Yampil Region, Vinnitsa Oblast

The cemetery complex was excavated in 2014 and is located on the crest of a long promontory in a north-west to south-east direction. From the western border with the Dniester Valley, about 1 km away from the corridor of this river, to the east of the valley of the Markovki. The most important point of this zone is Barrow mound number IV; its height at the commencement of excavation work was measured at about 2.4 m and the diameter at 35 m. About 80 m to the south along a line of a north-east to west-south axis three small mounds were situated (barrows I-III), whose diameter was measured at about 20 to 24 m and the preserved height merely at 0.15 to 0.3 m. The limitations of the surrounding terrain only allowed for the investigation of but one central and western part of barrow IV. Here the smaller mounds were identified entirely.

Within tumulus 1 four features were identified. One of the features was the hypothetical central grave, in which no traces of inhumation alas, were recorded – only in its fill did a fragment of ceramic ware and wood that were identified allow to categorize this grave to the Gordineşti group, Tripolye culture. The chronology of the other two features can be linked to the Iron Age. For the purposes of DNA analysis samples from the fourth feature were taken, which was sunk into the eastern part of the mound.

Barrow 1, Grave 4 (Catacomb culture) – poz220 and poz221

This is a double grave of the Catacomb culture. In the pit shaped oval and measuring 2.2×1.5 m, oriented south-north, on the western side a woman was laid to rest aged around 15 and on the eastern side a man aged 35-50. The only element of furnishing was a stone club with the remains of a wooden handle and copper mounting, placed at the shoulder of the deceased male.

Under tumulus mounds II and III seven features in all were found, which point to the fact that those responsible for construction were of Eneolithic communities and that subsequent phases of funerary exploitation were undertaken after the Bronze Age. No materials were gained from these graves for the purposes of DNA marking.

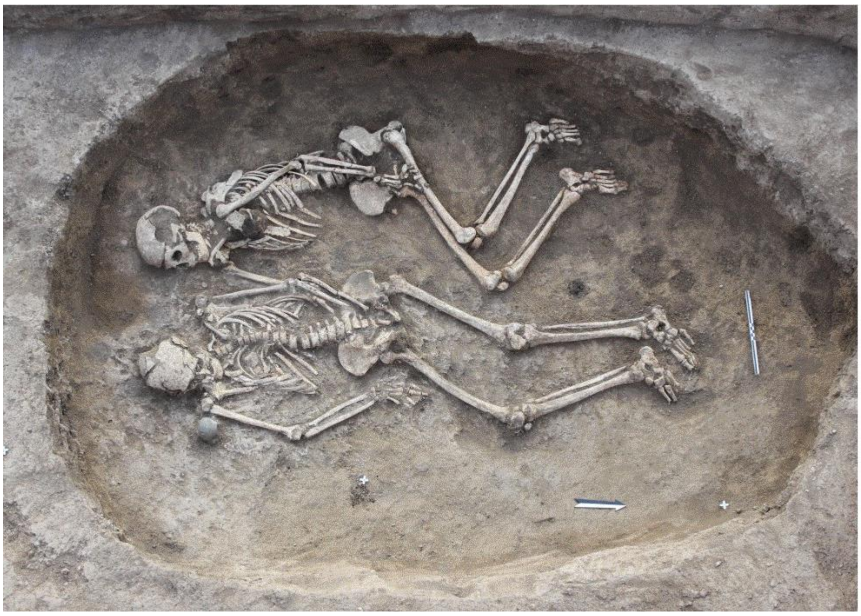

Supplementary Fig S11. Grave 4 (skeleton 1 and 2), Prydnistryanske barrow 1.

The initial, small mound of tumulus IV, measuring a diameter around 25 m was raised over Eneolithic feature of the Tripolye culture. From the subsequent two stages of its enlargement there originate inhumations of the Yamnaya culture from which samples were taken for DNA analysis – features 4 and 6 from the older phase and 3, 8 and 9 from the younger phase of the Yamnaya culture. The final results of these analyses provided DNA from skeletons from graves 4, 8 and 9.

Barrow IV, Grave 4 (Yamnaya culture) – poz222

The male interred in feature 4 died, aged 35-50. The deceased lay in a pit, measuring 0.9×2.85 m, in a supine position with raised knees and both arms along his body. Part of the furnishings entailed a mat of organic materials upon which the deceased rested and a small lump of ochre placed by the skull (Supplementary Figs S12, S13). The grave was secured by a wooden lid supported on stakes. In the upper part stone blocks were laid, thereby creating a slab measuring about 2.7×1.9 m that was in addition secured by an organic mat.

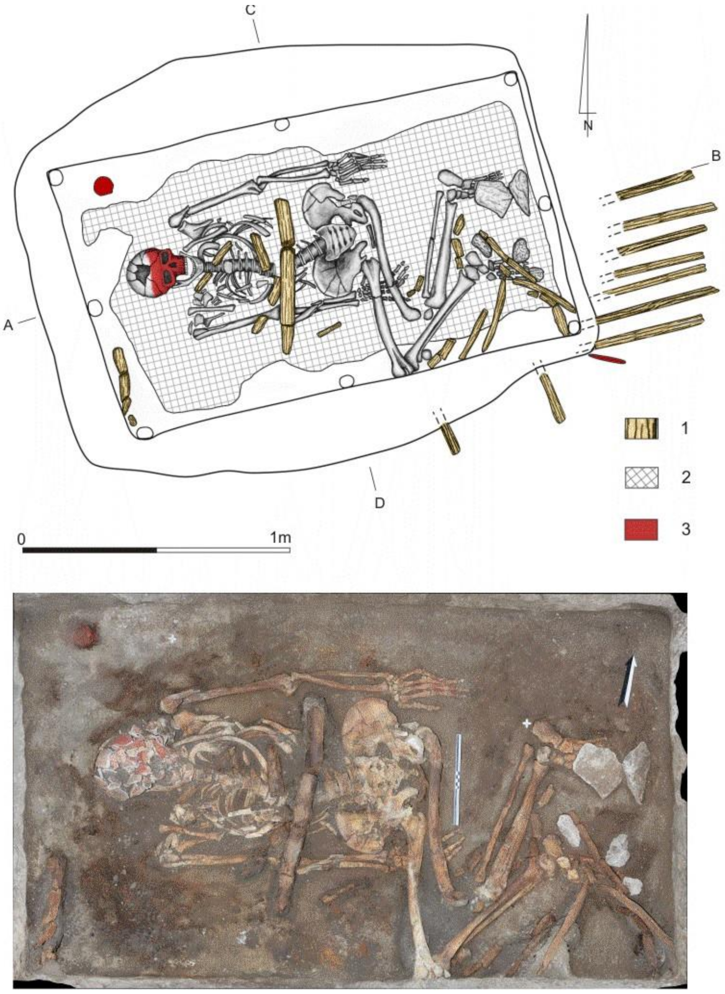

Supplementary Fig S12. Grave 4, Prydnistryanske barrow IV.

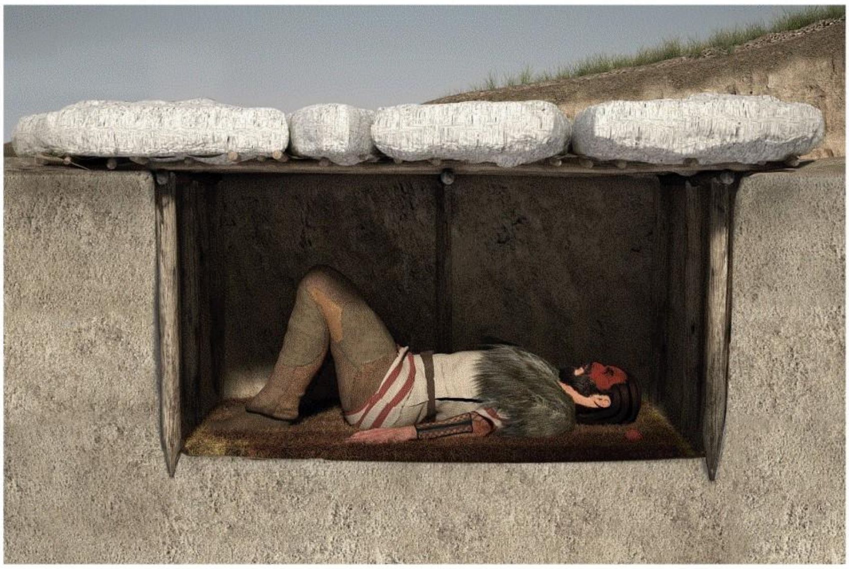

Supplementary Fig S13. Grave 4, Prydnistryanske barrow IV.

Barrow IV, Grave 8 (Yamnaya culture) – poz224

From the younger phase of tumulus enlargement by a community of the Grave Pit culture there originates grave 8 from the southern part of the tumulus. In the pit, measuring 1.6×1.3m, a male was placed in a supine position, aged 35-50 (Supplementary Fig S14). Elements of grave furnishings for the deceased were a knife blade found near the pelvis, small lumps of ochre and preserved traces of an organic mat under the skeleton. The grave was secured by a wooden lid.

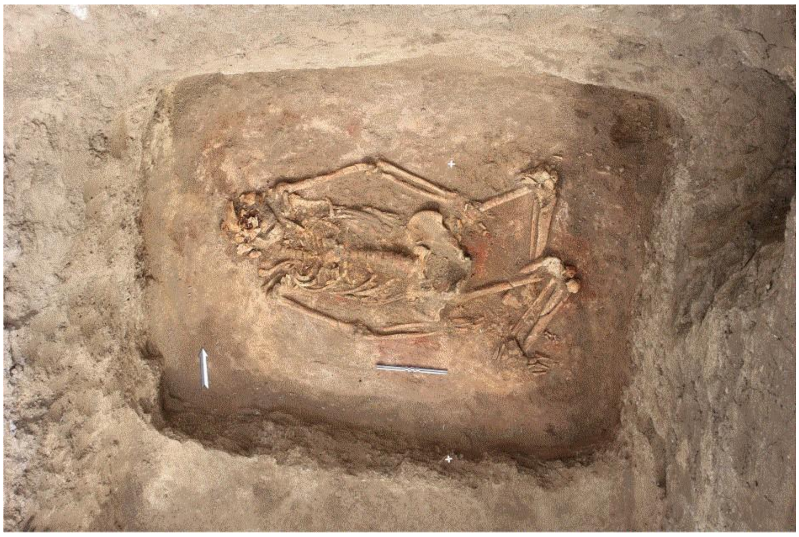

Supplementary Fig S14 Grave 8, Prydnistryanske barrow IV.

Barrow IV, Grave 9 (Yamnaya culture) – poz225

The pit of feature nine had very similar dimensions to that of grave 8 (1.85 x 1.4 m). The deceased male at the time of being laid to rest was aged 25-35 (Supplementary Fig S15). The deceased lay on a mat and was furnished with ochre. An animal bone was also found in the grave, while the upper part of the pit was covered by oak planks.

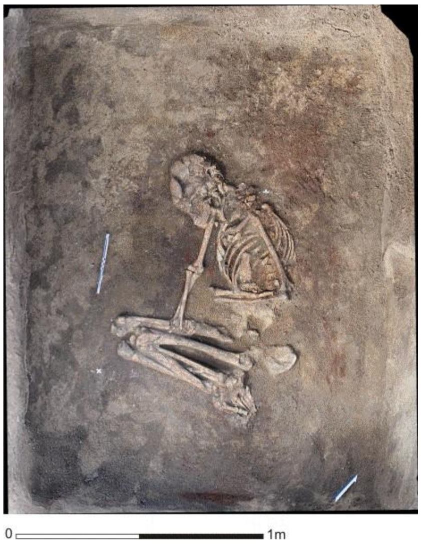

Supplementary Fig S15. Grave 4, Prydnistryanske barrow IV.

Corded Ware Culture individuals from Poland

In the period approximately 2800-2400 BC the system of cultural beliefs and patterns known as the Corded Ware culture characterized communities inhabiting the vast stretches of Europe. The eastern and southern part of this territory neighbored onto another great phenomenon in the third millennium BC – the Yamnaya culture. The regions of Małopolska also belonged to the broadly understood above mentioned zone, where relatively comprehensive grave finds originate (from Barrows and flat cemetery complexes). In this territory specific forms of funerary rites have been registered (among others catacomb graves), which could testify to ties with the Steppe culture circle of the north-west Black Sea region. The significance of this relationship is confirmed by some artifacts in the form of grave furnishings.

Małopolska Corded Ware culture sites can be said to have created several settlement concentrations located in the upland zone on territories covered with loess soils. The largest and best researched such concentration is in the western part, located on the left bank of the Vistula from where the graves presented below originate in Książnice and Malżyce. Other concentrations can be found in the Carpathian zone, Lublin Upland and the western part of the Volhynia Upland. It is the latter where the barrow from Hubinek it is located.

Hubinek, Ulhówek commune, district of Tomaszów Lubelski, site 2, barrow 1

The site is located amidst the concentration of Corded Ware culture barrows in the Sokal Hills, on the culmination of a loess rise. In 2011 the western and middle part of a large mound (about two thirds of its surface) was investigated, partially leveled as a result of farming practices. Under the mound two circular small ditches were discovered, the smaller located in the northern part, surrounding grave 9. The larger of the two surrounded feature 5 was most likely related to the second phase of mound construction and its significant enlargement. In the southern part of the barrow two human graves were subsequently excavated (features 4 and 8) and the inhumation of a horse (Feature 7).

In terms of funerary rites and their associated traits the finds from Hubinek differ in respect to discoveries from the south-east of Poland, which are clearly different from the numerous graves of the Corded Ware culture located in the Sokal Hills[3]. Their distinguishing characteristic is the non-ceramic nature of the inventory and the use of ochre to sprinkle the body of the deceased. Radiocarbon dating points to a relatively early age for grave 9 (3025 – 2898 BC, Supplementary Table S1).

Grave 4 (Corded Ware culture) – poz235

The pit grave was sunk into the southern part of the mound and was in the shape of a rectangle, measuring 175 x 100 cm, and along a west-east axis. Within it a rectangular wooden construction was distinguished, measuring about 130 x 55 cm and traces of planks with a diameter of about 5cm. At its bottom there lay the remains of two individuals aged *infans II* (Supplementary Fig S16*)*, placed in an antipodean position, most likely crouched (the anatomical arrangement was highly disturbed). Traces of ochre were identified on the bones and the grave had no furnishings.

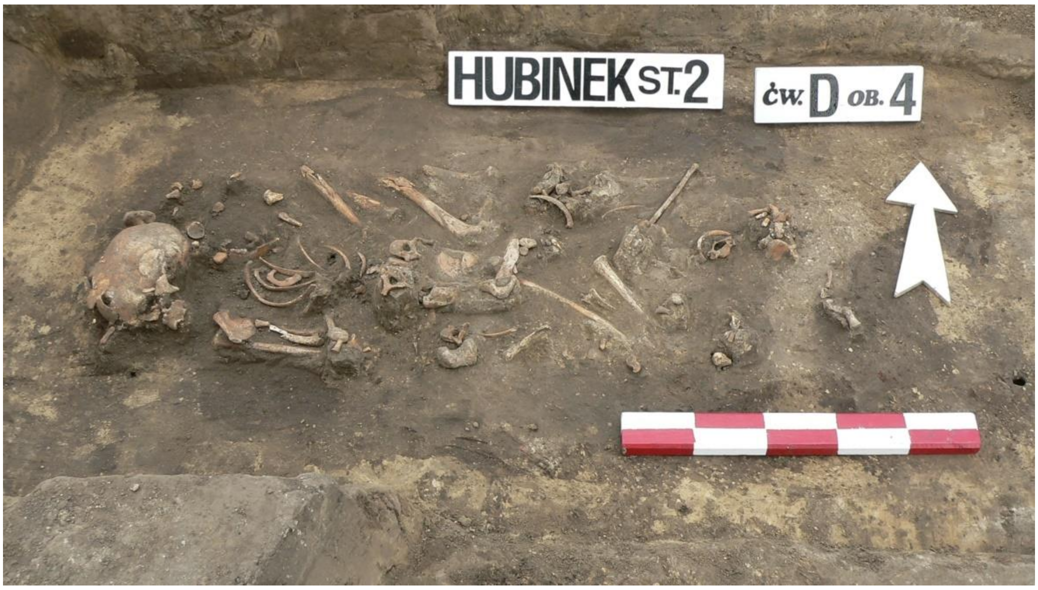

Supplementary Fig S16. Grave 4, Hubinek site 2, barrow 1.

Grave 8 (Corded Ware culture) – poz234

The pit grave was sunk into the southern part of the mound along a west-east axis and had a shape similar to a rectangle with rounded maculate corners, measuring 210 x 120 cm, with smudges of ochre with which the skeleton was also covered. The grave of the male (?), aged 25-30 rested at the bottom of the pit (Supplementary Fig S17), in a supine position with the head and lower limbs turned to the right. Both the bones and the bottom of the pit were profusely sprinkled with ochre, and the grave had no furnishings.

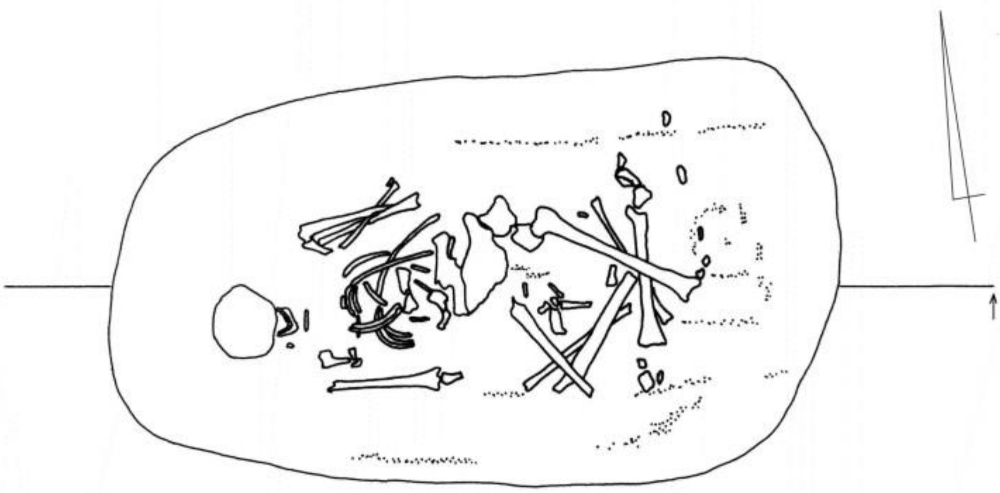

Supplementary Fig S17. Grave 8, Hubinek site 2, barrow 1.

Grave 9 (Corded Ware culture) – poz232

The pit grave in the northern part of the barrow, surrounded by a circular small ditch, ran along a west-east axis. It was in the shape of a rectangle with rounded corners, measuring 235 x 140 cm. At the bottom the skeleton of a male aged 20-25 was found, placed in a 'side 'position with the lower limbs contracted, towards the left side (Supplementary Fig S18).

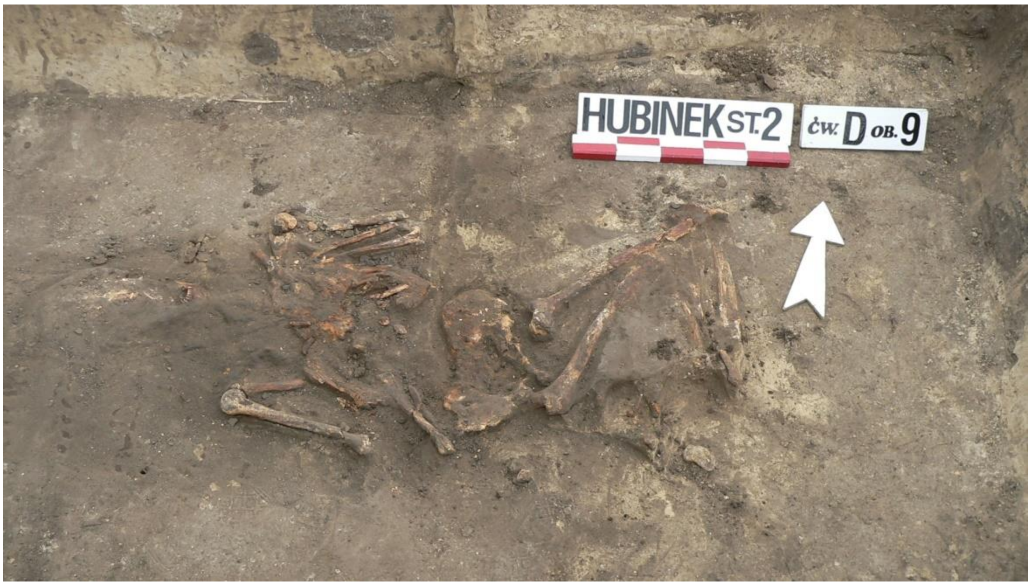

Supplementary Fig S18. Grave 9, Hubinek site 2, barrow 1.

Malżyce, Czarnocin commune, district of Kazimierza Wielka, site 30, barrow 1

The site is located in the Nida Trough, within the western Małopolska loess rise, which contains a cemetery complex that arose at barrows and long burial mounds of the megalithic Funnel Beaker culture. In the period 2004-2005 barrow 1 was investigated – entirely leveled as a result of intensive farming practices. The circular shape of the mound, measuring about 10-12 m in diameter, was marked only by a relevant darkening of the ground surface. In its centre a grave of the Final Beaker culture was discovered[4]. From the south-east, features of the Corded Ware culture were sunk into the mound: graves with a niche construction (nos. 2, 4 and 7), pit grave (no. 5) and a small pit with a vessel (no. 3). Moreover, at the edge of the mound, three inhumations of the Mierzanowice culture (nos. 8-10) were found. In respect to the construction traits of graves the placement of burials and characteristics of furnishings, the features from Malżyce represent a specific Małopolska variant of the Corded Ware culture known as the Kraków-Sandomierz group[5,6].

Grave 2 (Corded Ware culture) – poz279

The niche grave was sunk into the central part of the barrow. The niche measured 280 x 220 cm. At its bottom (135 cm) a richly furnished inhumation of a male aged adultus/maturus was discovered (Supplementary Fig S19). The deceased was placed on his back with the lower limbs turned to the right. Only several teeth represented remains of the skull. The grave furnishing included a beaker, stone axe, flint artefacts (axes, 6 projectile points and two knife blade insets), stone polishing slab and

a copper punch. The radiocarbon dating, taken from the bones in the grave reads: 3860±35 BP (Poz-59407), that is 2454-2236 BC.

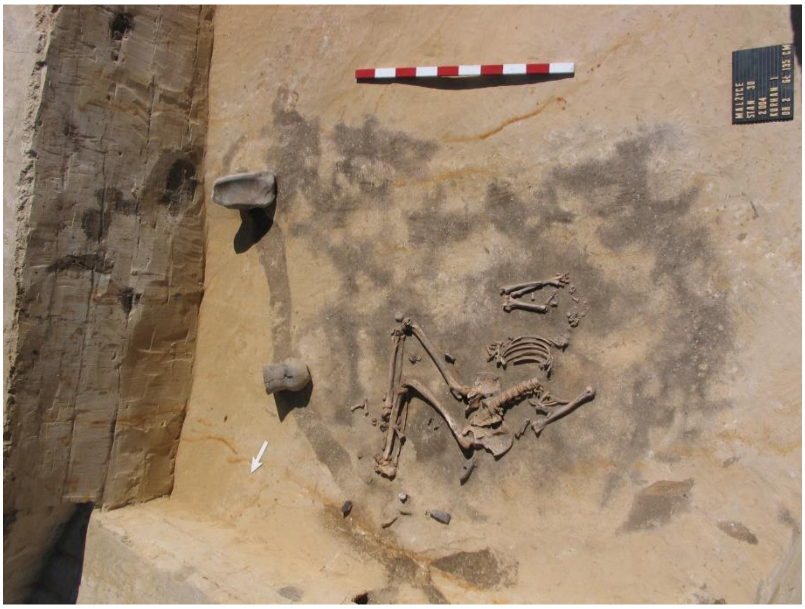

Supplementary Fig S19. Grave 2, Malżyce site 30, barrow 1.

Grave 4 (Corded Ware culture) – poz280

Feature with a niche construction sunk into the peripheral part of the barrow. The niche measured 190 x 175 cm and a depth of 145 cm. At its bottom there lay the skeleton of a female aged maturus/senilis, placed on the left side along a west-east axis (Supplementary Fig S20). By the skeleton a ceramic jug was discovered as well as two flint artefacts (axe and flake tools), whetstone and a bone awl.

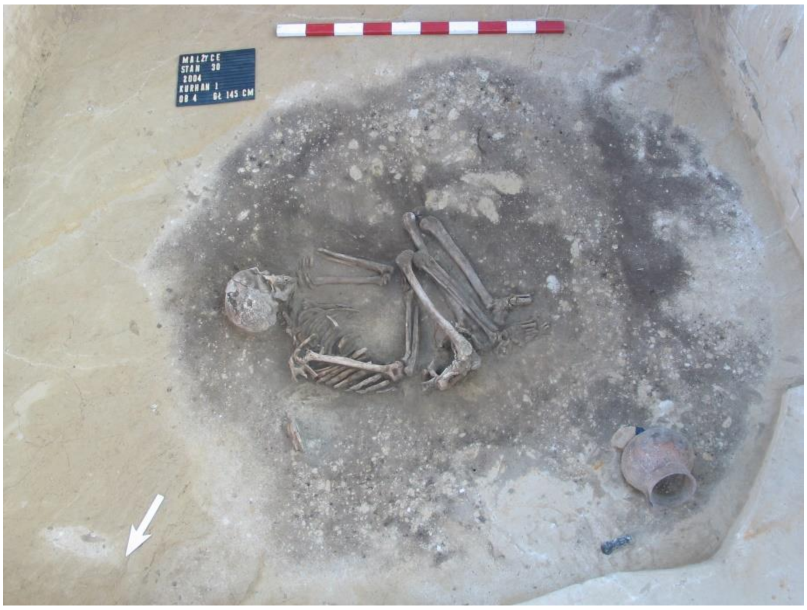

Supplementary Fig S20. Grave 4, Malżyce site 30, barrow 1

Grave 5 (Corded Ware culture) – poz281

In a pit measuring 95 x 70 cm and a depth of 45 cm a poorly preserved skeleton of a child aged infans I/II was discovered (Supplementary Fig S21). The deceased was placed in a crouched position, most likely on the left side. A small beaker was found at its side.

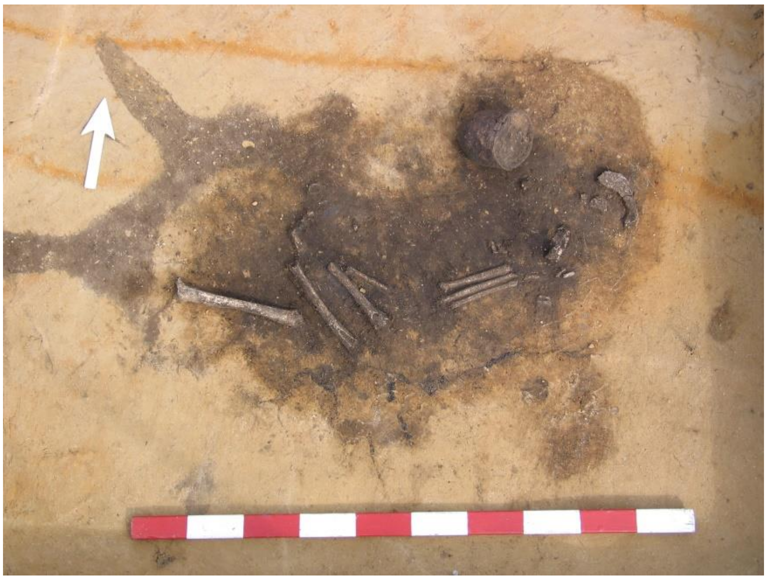

Supplementary Fig S21. Grave 5, Malżyce site 30, barrow 1.

Grave 7 (Corded Ware culture) – poz282

A niche grave discovered at the edge of the tumulus. The niche had an oval shape and measured 205 x 145 cm. At its bottom (105 cm), an adult deceased individual was found, sex unknown (Supplementary Fig S22). The deceased was placed on their back, head and lower limbs turned to the left. Grave furnishings included a ceramic beaker, flint axe, a bone awl and five flint flakes.

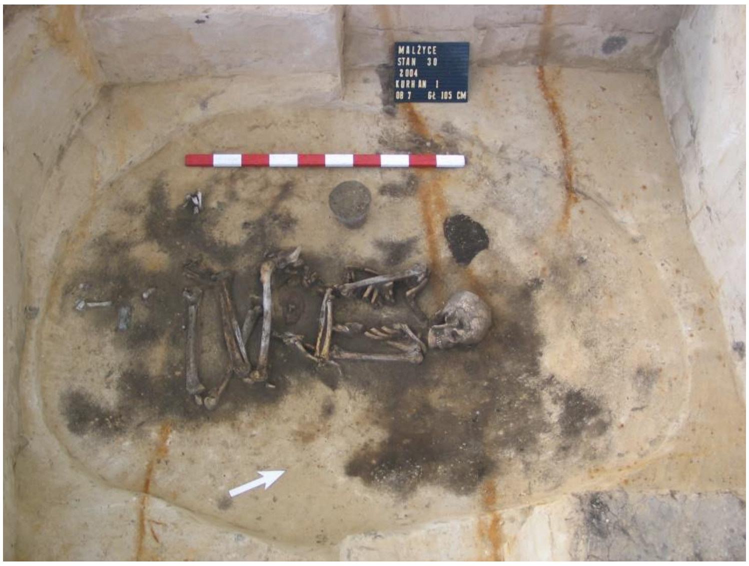

Supplementary Fig S22. Grave 7, Malżyce site 30, barrow 1.

Książnice, Pacanów commune, district of Busko Zdrój, site 2

The site in Książnice, located in the eastern end of the Pinczów Hummock, covers the peak of a small rise (200.15 m.a.s.l.), overlooking from the south and south-west over the valley of a nameless waterway.

During excavation research that had begun in 2001, conducted by Stanisław Wilk, a vast Neolithic and early Bronze Age sepulchral settlement complex was identified. The above complex contained a vast cemetery of the Lublin-Volhynia culture, Funnel Beaker Culture settlement, Złota culture cemetery complex and settlement, Corded Ware culture cemetery complex as well as a Mierzanowice culture cemetery complex and settlement.

The Kraków-Sandomierz group cemetery complex, Corded Ware culture contains nine graves, four of the first creating a concentration located in the southern part of the site and the other five were discovered in its eastern part. The graves subject to analysis, nos.1 and 2, were discovered in July and August respectively in 2003.

Grave 1 (feature 2/03) (Corded Ware culture) – poz287

A pit grave in the shape of an elongated oval along a north-south axis with a thickness of about 20 cm. At a depth of 60 cm a skeleton was found of a child (Infans I) placed in a crouched position on its back, the skull pointing north (Supplementary Fig S23). The grave furnishings included a flower pot beaker that was embellished in its upper section with horizontal cord imprints and placed in the southern part of the pit as well as five small flint products: a Volhynia flake, two chips (from Volhynia and Świeciechów flint), two fragments of flint splinters (from Volhynia and Jurassic flint) that were found in the fill of the pit grave.

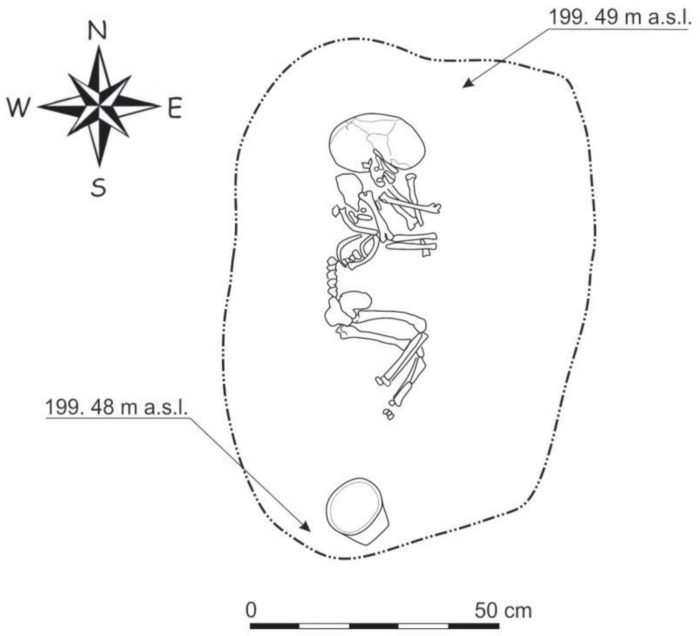

Supplementary Fig S23. Grave 1, Książnice site 2.

Grave 2 (feature 3 and 3a/03) (Corded Ware culture) – poz286

The grave was of a niche construction placed in a west-east axis with a rectangular entrance shaft (feature 3/03) and an oval niche grave along a north-south axis (feature 3a/03). In the western part of the shaft the entrance to the niche was obscured by a slab of loess. The niche was filled with a black-brown humus and did not bear any signs of collapse.

At the bottom of the niche grave, at a depth of 125 cm, there lay a well preserved skeleton of an adult female, placed along a north-south axis in a crouched position on its left side with highly contracted lower limbs and the skull pointing north (Supplementary Fig S24). The grave furnishings contained three clay amphorae placed in the southern part of the grave, of which two (double-ear and quadruple-ear) are of middle size with necks embellished with horizontal cord imprints, while the third – greater in size with a spherical belly, boasts six vertically attached ears and necks and an upper part of the belly covered with three rows of imprints bearing a vertical small stamp. Apart from the vessels, the grave revealed a semi-product of a bone awl and two entire awls, a small polishing stone made out of red sandstone and a four-sided axe made from Świeciechów flint. Moreover, the niche grave fill revealed nine flint products: seven flakes (from Volhynia, Jurassic and Chocolate flint), a Świeciechów flint knife blade insert with retouched convergent sides and a Volhynia flint unidirectional flake splinter.

On the basis of the recorded description of the funerary rite and typological analysis of artefacts found, the above described graves ought to be dated to the decline of the Corded Ware culture on the Małopolska Upland, c. 2400-2300 BC.

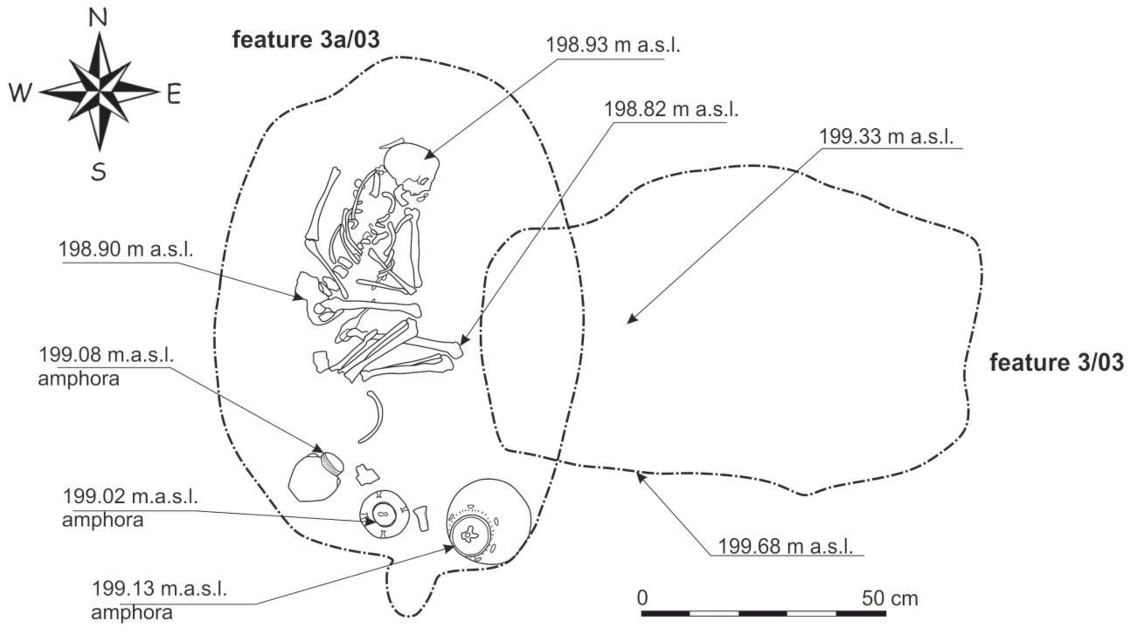

Supplementary Fig S24. Grave 2, Książnice site 2.

Graves 1 and 2 of the Moravian Corded Ware Culture, Držovice site, the Prostějov region, Czech Republic

During the rescue research (2014-2015) preliminary to the construction of 25 family houses, a part of the bi-ritual burial site of the Moravian CWC culture was found in the village Držovice (site called Pastviska) in the Prostějov region (middle Moravia), with 6 graves at considerable distances from each other (interval 17-48 m). The burials were located on a moderate SW slope above the Český potok brook terrace (100-200 m) at an altitude of 220-228 m[7].

The two graves were found 17 m apart, composing part of a group burial ground. The burials can generally be dated to the period of local development of Moravian CWC, which in absolute terms represents the period between 2600/2500 - 2300/2200 BC.

Grave 1 (H 1/ob. 570) (Corded Ware culture) – poz256

Grave 1 represented an oblong burial pit (197 x 104 x 50 cm) with markedly rounded corners (Supplementary Fig S25). The skeleton on the bottom belonged to an adult individual aged 40 to 50 years[8] at the time of death with a stature of about 171.88 +/-4.49 cm[9]. Estimation of the biological sex according to the morphology of the skeleton was not available due to bad preservation and low completeness of the sex specific skeletal parts (hip bone, skull). The individual was lying on his/her right side and clear evidence of secondary manipulations with bones was recorded based on bones scattered within the grave filling and bottom. The burial was gifted/accompanied with a ceramic jug of the Balkan type (so called variant Alsónémedi - F1/2, see[10], a bowl of the Moravian type (Hm), an egg-shaped pot (P), a cup-shaped mug (SN), a bone awl, a small copper awl (?) and animal bones. The grave can be attributed to very late phase of the local CWC (phase IIIb/c).

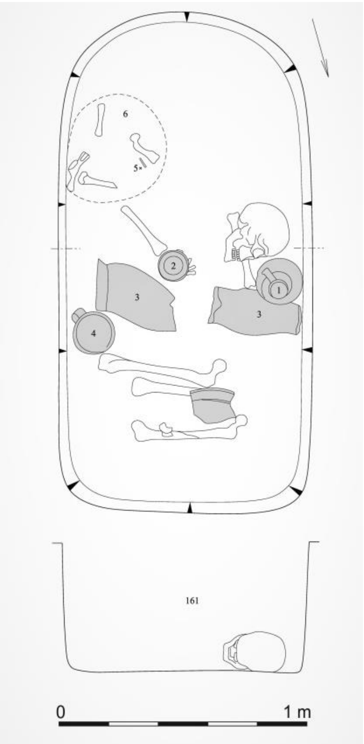

Supplementary Fig S25. Grave 1, Držovice site, Czech Republic.

Grave 2 (H 2/ob. 571) (Corded Ware culture) – poz257

Grave 2 was formed as a regular rectangle with rounded corners and straight walls (179 x 129 x 100 cm) with NE-SW orientation. At the bottom a well-preserved skeleton of an adult man[11] aged 35-40 years old in the time of death[8] with a stature estimated to 171.88 +/-4.49 cm[9] (femur length) was found. The body was lying in a strongly crouching position on the right side and with the head oriented to SE direction and face turned down. In front of the body a large jug of the Dřevohostice-type (CD1) with a bulbous body and a pair of plastic ribs (ripH) on a wide tape ear was placed. The body of the jug up to the maximum bulge was provided with a weak decorating with straw imprints. Behind the feet of the skeleton a tall cup with imprints of left-twisted cord (13x) on its throat with straw decoration similar to the mention above was found. Next to the jug a bone awl was found. Behind the body, the remnants of a stone ax of a simple shape (OHA) were placed and a flint blade with double-sided retouching was found next to the pelvis. It is therefore a typical man's grave equipment (grave goods). The findings in this is grave seems to be slightly older than those in the grave 1 and can be dated into the local phase IIIa.

References:

1. Klochko, V. I. & Kośko, A. The Societies of Corded Ware Cultures and Those of Black Sea Steppes (Yamnaya and Catacomb Grave Cultures) in the Route Network Between the Baltic and Black Seas. in *Baltic-Pontic Studies* **14,** 269–301 (2009).
2. Kośko, A. Podolia as a Cultural Contact Area in the 4th/3rd-2nd Millennium BC. *Baltic Pontic Studies* **20,** (2015).
3. Machnik, J., Bagińska, J., Koman, W., Libera, J. & Gazda, L. *Neolityczne kurhany na Grzędzie Sokalskiej w świetle badań archeologicznych w latach 1988-2006*. (Polska Akademia Umiejętności, 2009).
4. Tunia, K. & Włodarczak, P. Barrow of the Funnel Beaker Culture in Malżyce, Kazimierza Wielka district. *Spraw. Archeol.* **63,** 203–219 (2011).
5. Machnik, J. *Studia nad kulturą cermiaki sznurowej w Małopolsce*. (1966).
6. Włodarczak, P. *Kultura ceramiki sznurowej na Wyżynie Małopolskiej - Instytut Archeologii i Etnologii PAN*. (2006).
7. Fojtík, P. Držovice (k.ú. Držovice na Moravě, okr. Prostějov),. *Přehl. Výzk. Brno* 186–187 (2016).
8. Lovejoy, C. O. Dental wear in the Libben population: its functional pattern and role in the determination of adult skeletal age at death. *Am. J. Phys. Anthropol.* **68,** 47–56 (1985).
9. Sjøvold, T. Estimation of stature from long bones utilizing the line of organic correlation. *Hum. Evol.* **5,** 431–447 (1990).
10. Peška, J. & Králík, M. “Nagyrév Jugs” and Their Archaeological Context. in *Transition to The Bronze Age – Interregional Interaction and Socio-Cultural Change in the Third Millenium BC Carpathian Basin and Neighbouring Regions.* 245–286 (Archaeolingua Alapítvány, 2013).
11. Murail, P., Bruzek, J., Houët, F. & Cunha, E. DSP: A tool for probabilistic sex diagnosis using worldwide variability in hip-bone measurements. *Bull. Mém. Société D’Anthropologie Paris* 167–176 (2005).

Methods

Ancient DNA analysis – mitochondrial genome capture

Preparation of biotinylated probes

Biotinylated probe DNA was generated by LR-PCR (PrimeSTAR GXL DNA Polymerase, Takara Bio) according to the manufacturer's protocol. The whole mitochondrial genome was amplified in multiple 25μl LR-PCR reactions using the two primer sets L06363/H14799 (8476 bp) and L14759/H06378 (8340 bp) following[1]. PCR reactions were pooled for each product and loaded onto 1% Agarose gels. Bands of the correct size were visualized with UV light and exercised with a clean scalpel blade. The gel slices were weighted and the DNA was extracted with the use of QIAquick Gel Extraction Kit (QIAGEN) following manufacturer's instructions. The concentration of the cleaned LR PCR products was than measured using NanoPhotometer™ Pearl (Implen). Acquired DNA fragments were pooled in equimolar amounts and adjusted to 20ng/μl. The DNA was then sheared on a Bioruptor Plus sonication device (Diagenode) for 120 cycles (30s on /30s off) with the output selector switched to (H)igh. The resulting fragmented DNA (∼100–200 bp average size, range 100–500) was subjected to end repair and dA-tailing by a NxSeq® AmpFREE Low DNA Library Kit (Lucigen) according to the manufacturer's protocol. Ligation was also performed with this kit, but with custom adapters prepared following[2]. Three microliters of 1:10 dilution of this T7 adaptor stock was used for the ligation reaction, according to the library preparation kit instructions. The libraries were then cleaned and size selected using AMPure® XP Reagents (Agencourt-Beckman Coulter) following the library preparation kit instructions, using 0.95x and 0.85x beads of original sample volume for size selection step, in order to select fragments ∼150–350 bp in length. The size selected libraries were PCR amplified in ten separate reactions with the following components: 5 μl 2× HiFi HotStart ReadyMix (KAPA), 20 μl H_2O, 1 μl PCR, same as T7 oligo 1[2] , and 4 μl purified ligation mix. The cycling conditions were as follows: 98°C/3 min, 98°C/20 s; 18 cycles of 60°C/15 s, 72°C/30 s; 72°C/10 min. The reactions were pooled and purified with AMPure XP beads, eluting in 30 μl H_2O. The concentrations and DNA fragment length distributions of the cleaned PCR products, were calculated using High Sensitivity D1000 Screen Tape assay on 2200 TapeStation system (AgilentTechnologies).

The bait libraries were transcribed into biotinylated RNA following[2] with minor modifications. Between ∼200 and ∼300 ng of amplified library was used for the in vitro transcription. The reaction product was then purified with an RNeasy Mini kit (QIAGEN) according to the manufacturer's instructions, using the same 30 μl of H_2O twice for the final elution. The length distribution and concentration of the final RNA bait was than assessed using RNA Analysis ScreenTape on 2200 TapeStation system (Agilent Technologies).

References:

1. Brotherton, P. *et al.* Neolithic mitochondrial haplogroup H genomes and the genetic origins of Europeans. *Nat. Commun.* **4,** 1764 (2013).
2. Carpenter, M. L. *et al.* Pulling out the 1%: Whole-Genome Capture for the Targeted Enrichment of Ancient DNA Sequencing Libraries. *Am. J. Hum. Genet.* **93,** 852–864 (2013).

Statistical analyses

PCA for mtDNA hgs frequencies was calculated using Python 3.5 and Scikit-learn v. 0.18.1[1] (Scikit- learn Machine Learning) package. We utilized Matplotlib 1.5.1 Python package[2] for plotting the PCA results and mtDNA hgs loadings.

We have used a centroid-based clustering approach to examine the PCA results and search for logical clusters within our data. We applied the k-means method as implemented in Scikit-learn v. 0.18.1 Python package[1] to the first 5 principal components coming from the PCA analysis. To access the quality of the clustering, we computed the silhouette parameter for k-means clustering with $k = \{2, 3, 4, \ldots, 9\}$. For each k we ran the k-means algorithm 50 times and took the result with the best silhouette score. All k-means variants can be found in the Supplementary Material Text material (Supplementary Figure S27 and S28).

To further explore the relatedness of populations according to the mtDNA hgs frequencies, we have run the t-Distributed Stochastic Neighbor Embedding (t-SNE) analysis[3] as implemented in Scikit-learn (18.1) Python package. t-SNE is a modern dimension reduction method, which employs an iterative algorithm to visualize the high-dimensional data into 2D or 3D, retaining the local structure of the data while also revealing some important global structure (i.e. clusters). Following parameters were used in the t-SNE analysis: early_exaggeration: 4.0, learning_rate: 8, method: exact, metric: euclidean, n_iter: 500000, n_iter_without_progress: 500, perplexity: 5.0.

F_{ST} values were computed in Arlequin 3.5[4]. The total of 21 populations were used in F_{ST} and following AMOVA analyses, consisting only of ancient individuals with complete mt genomes (Table S3), using Nei's average number of pairwise differences[5] and 10,000 permutations to estimate the p-values. To visualize F_{ST} values we employed multidimensional scaling (MDS) analysis with the use of Python Scikit-learn 0.18.1 package[1].

We have used the traditional method of analysis of molecular variance (AMOVA)[6] to access the population division of the samples with complete mt genomes. We ran AMOVA as available in Arlequin 3.5 software package. Our main focus in this analysis lied on the CWC and Yamnaya populations. First, we have divided our populations into four groups: (a) Group 1 (Hunter-Gatherers): eastern (HGE), western (HGW), Baltic (HGBal) and northern (HGN); (b) Group 2 (Early Neolithic farmers): Linear Pottery Culture (LBK), Near Eastern Neolithic (NENE), late LBK; (c) Group 3 (Middle Neolithic/Bronze Age Central Europe): middle Neolithic (MNE), Unetice (UNC), Bell Beaker Culture (BBC), early Bronze Age Germany (EBAG), Bronze Age Hungary (BAHu), Globular Amphora Culture (GAC); (d) Group 4 (Middle Neolithic/Bronze Age Eastern Europe): Bronze Age Russia (BARu), Iron Age Scythians from Moldova and Ukraine (SCU), Srubnaya Culture (SRU). In the next step, we combined our populations of interest (CWW, CWPlM, CWBal, YAW, YAE) in turn with every group, and continued with combining of the two connected populations (CWW + CWPlM + CWBal, YAW + YAE) with all the groups. This approach allowed us to correctly assign the CWC and Yamnaya populations to appropriate groups.

References:

1. Pedregosa, F. *et al.* Scikit-learn: Machine Learning in Python. *J. Mach. Learn. Res.* **12,** 2825–2830 (2011).
2. Hunter, J. D. Matplotlib: A 2D Graphics Environment. *Comput. Sci. Eng.* **9,** 90–95 (2007).
3. Maaten, L. van der & Hinton, G. Visualizing Data using t-SNE. *J. Mach. Learn. Res.* **9,** 2579–2605 (2008).
4. Excoffier, L. & Lischer, H. E. L. Arlequin suite ver 3.5: a new series of programs to perform population genetics analyses under Linux and Windows. *Mol. Ecol. Resour.* **10,** 564–567 (2010).

5. Nei, M. & Li, W. H. Mathematical model for studying genetic variation in terms of restriction endonucleases. *Proc. Natl. Acad. Sci. U. S. A.* **76,** 5269–5273 (1979).
6. Excoffier, L., Smouse, P. E. & Quattro, J. M. Analysis of molecular variance inferred from metric distances among DNA haplotypes: application to human mitochondrial DNA restriction data. *Genetics* **131,** 479–491 (1992).

Supplementary Table S6. Primer sequences used in the analyses.

Name	Primer sequences (5'→3')	Reference
IS4	AATGATACGGCGACCACCGAGATCTACACTCTTTCC CTACACGACGCTCTT	[1]
IS5	AATGATACGGCGACCACCGA	[1]
IS6	CAAGCAGAAGACGGCATACGA	[1]
Indexing* primer	CAAGCAGAAGACGGCATACGAGATxxxxxxxGTGACT GGAGTTCAGACGTGT	[1]
PISI	CCTCTCTATGGGCAGTCGGTGACCTACACGACGCTCT TCCGATCT	This study
AIS4	CCATCTCATCCCTGCGTGTCTCCGACTCAGCAAGCAG AAGACGGCATACGAGAT	This study

*"xxxxxxx" is 7bp index, one of 228 different indexes included in Mayer and Kircher (2010)

1. Meyer, M. & Kircher, M. Illumina sequencing library preparation for highly multiplexed target capture and sequencing. *Cold Spring Harb. Protoc.* **2010,** pdb.prot5448 (2010).

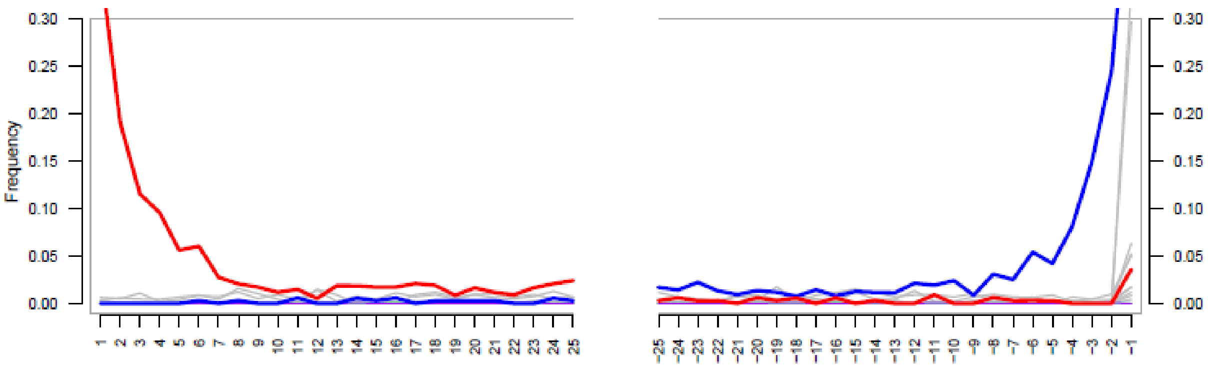

A. poz090

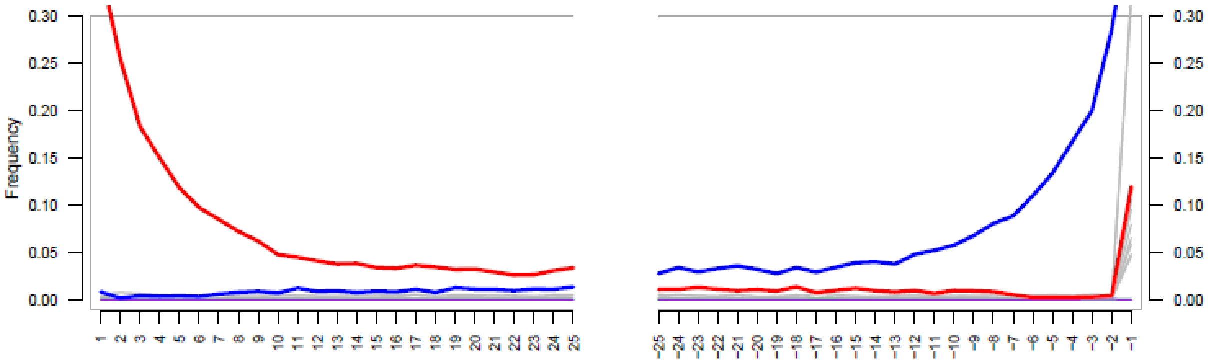

B. poz094

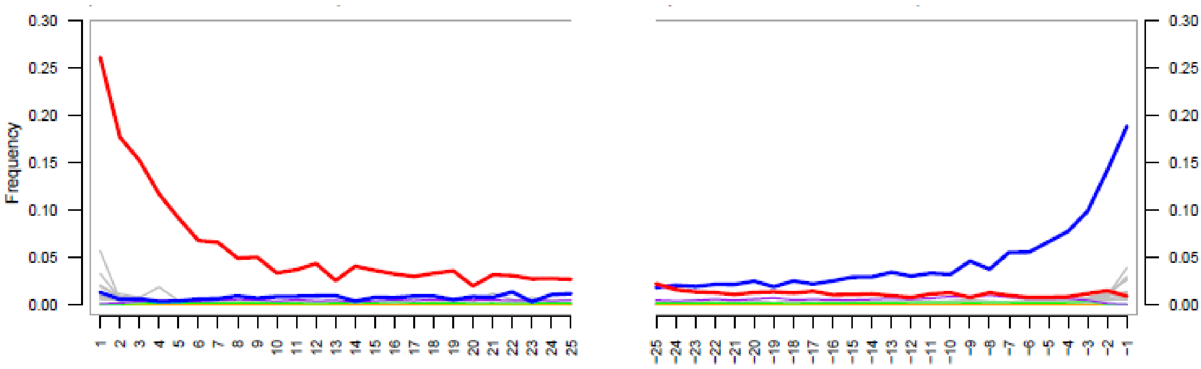

C. poz208

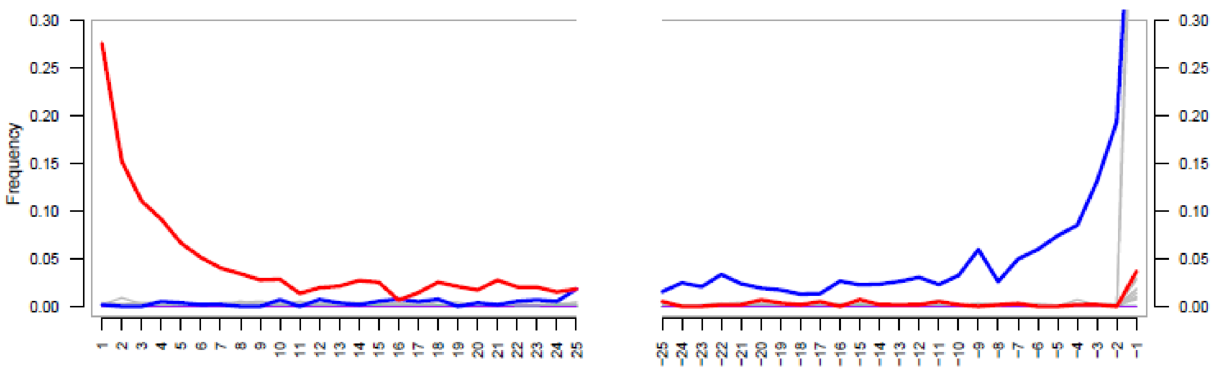

D. poz211

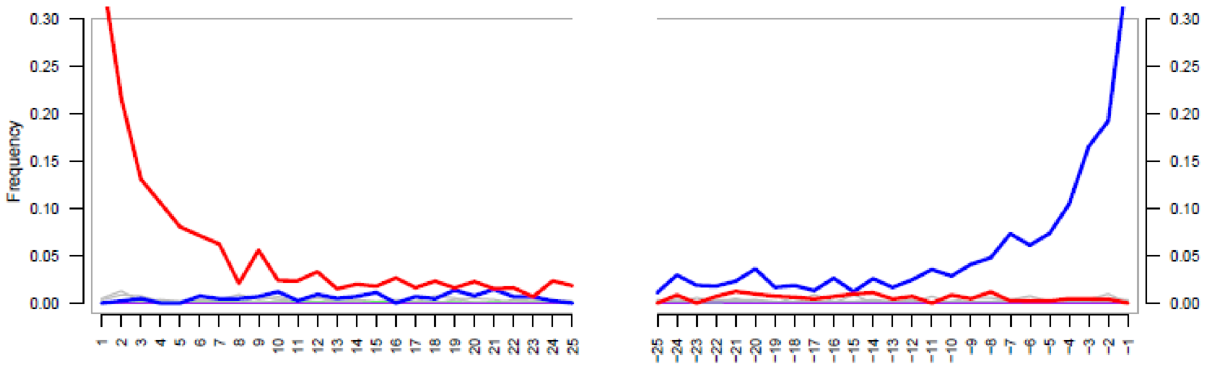

E. poz213

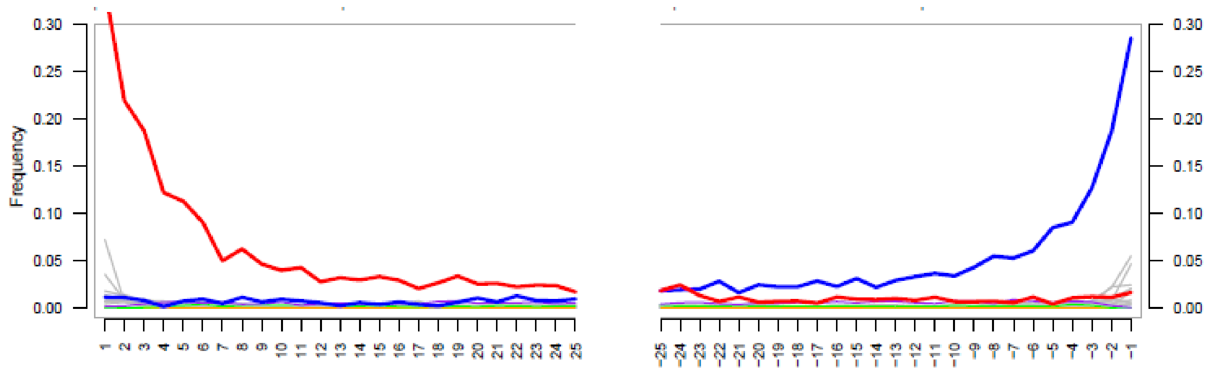

F. poz214

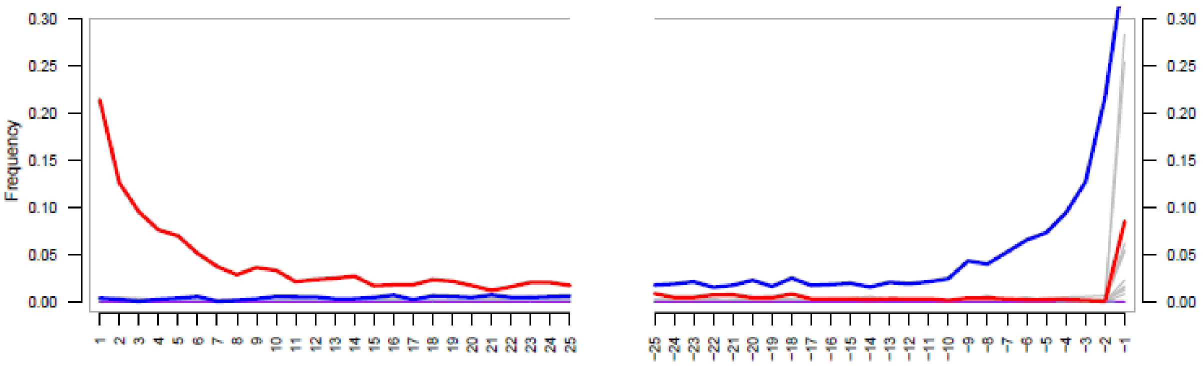

G. poz220

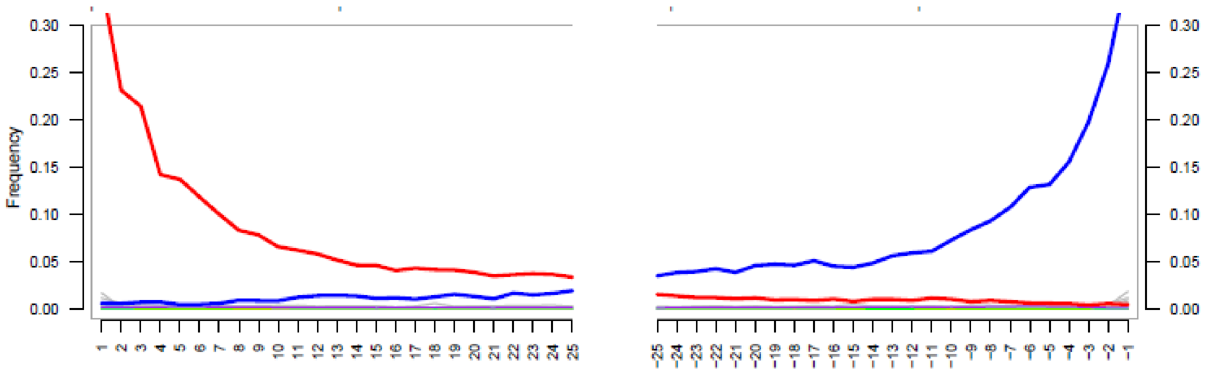

H. poz221

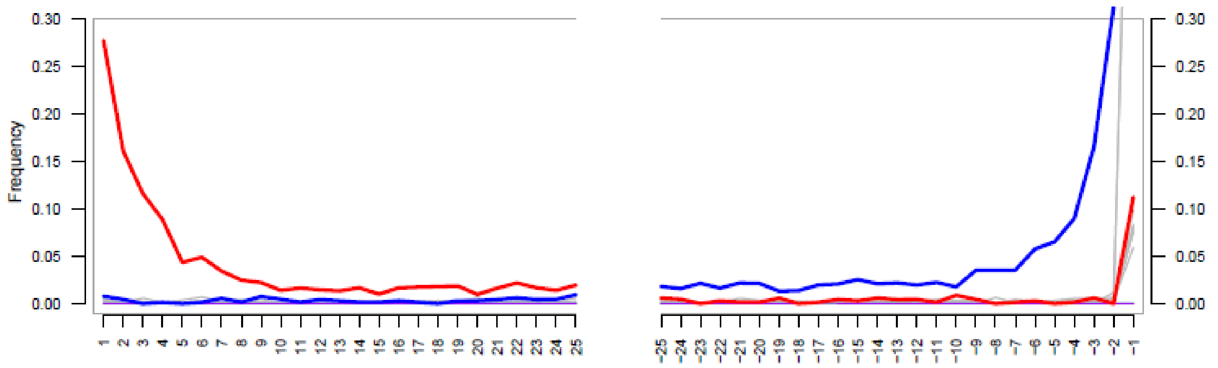

I. poz222

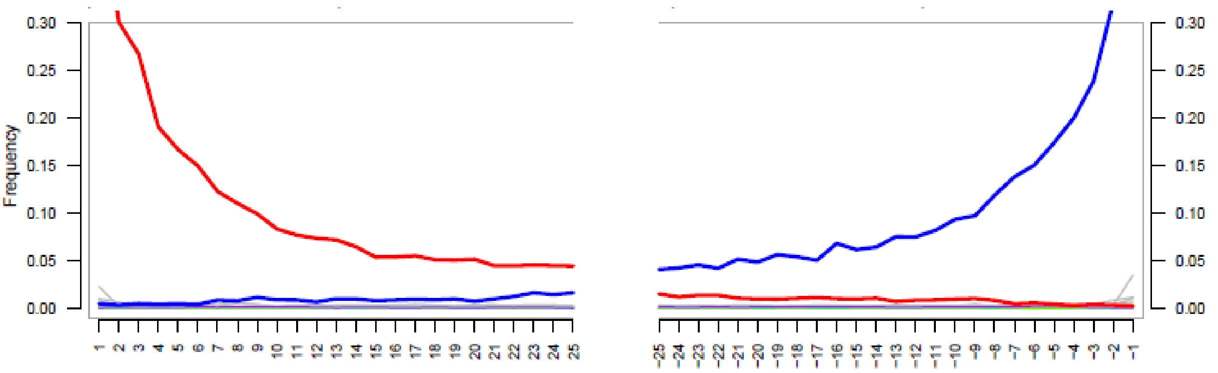

J. poz224

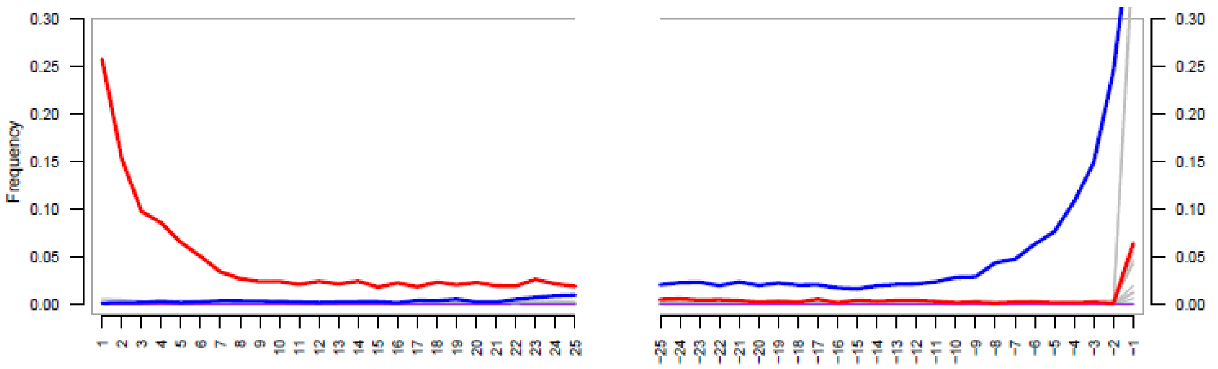

K. poz225

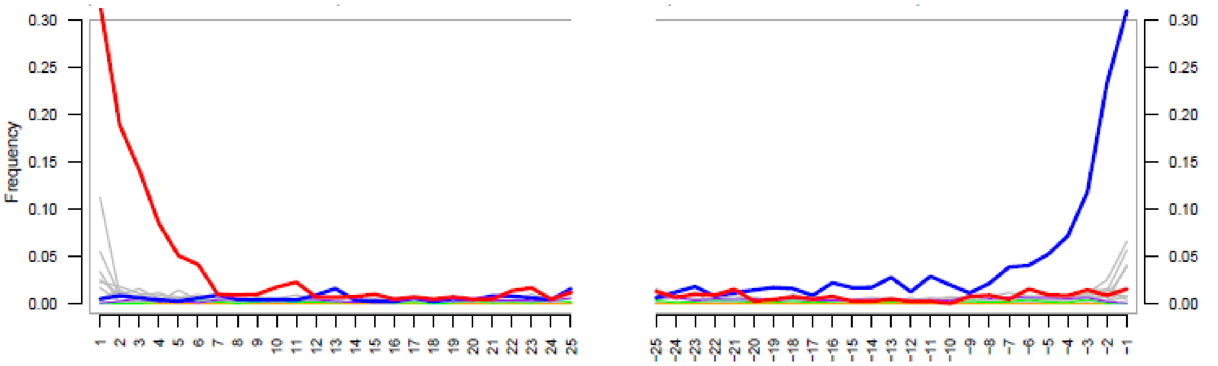

L. poz232

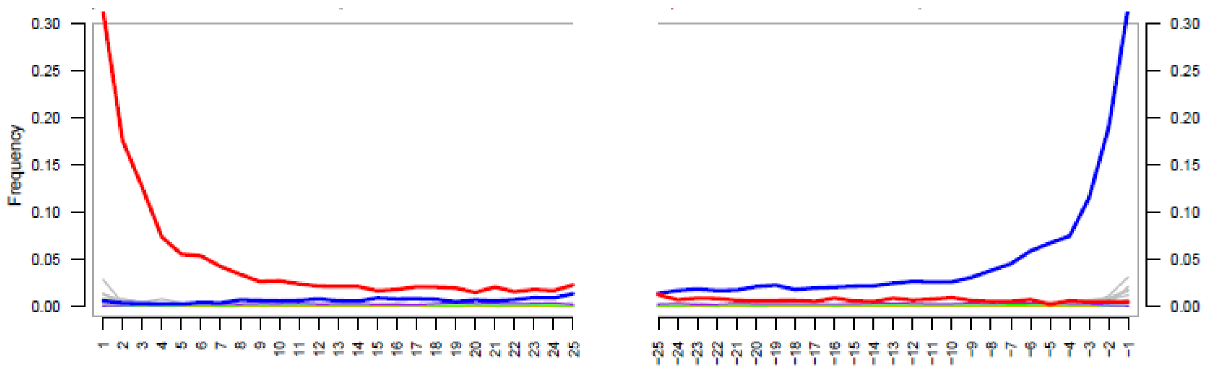

M. poz234

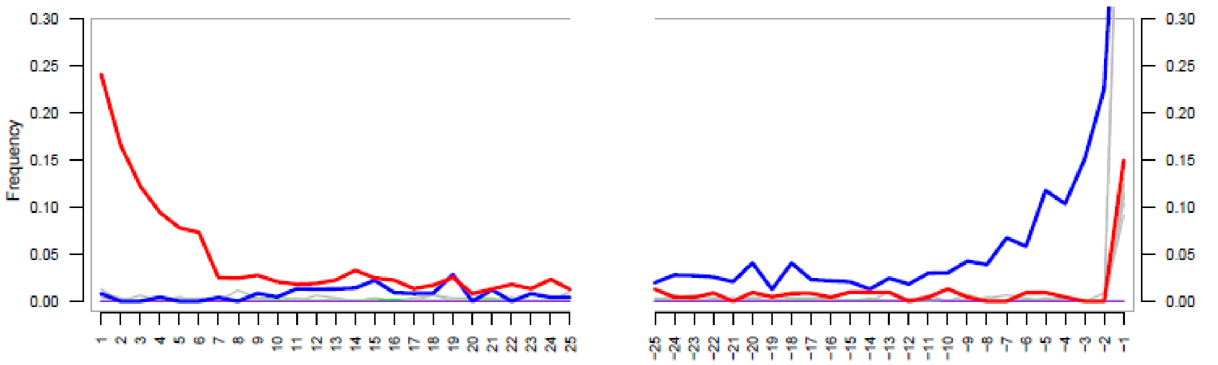

M. poz235

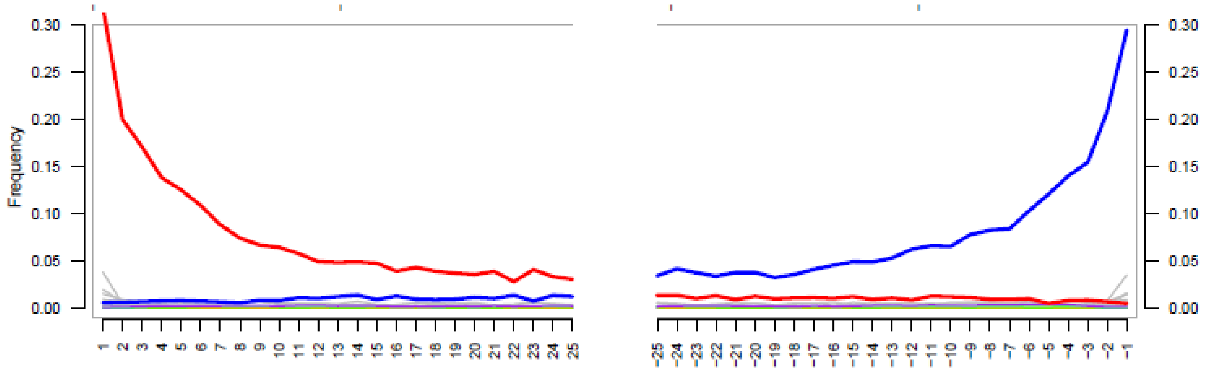

N. poz256

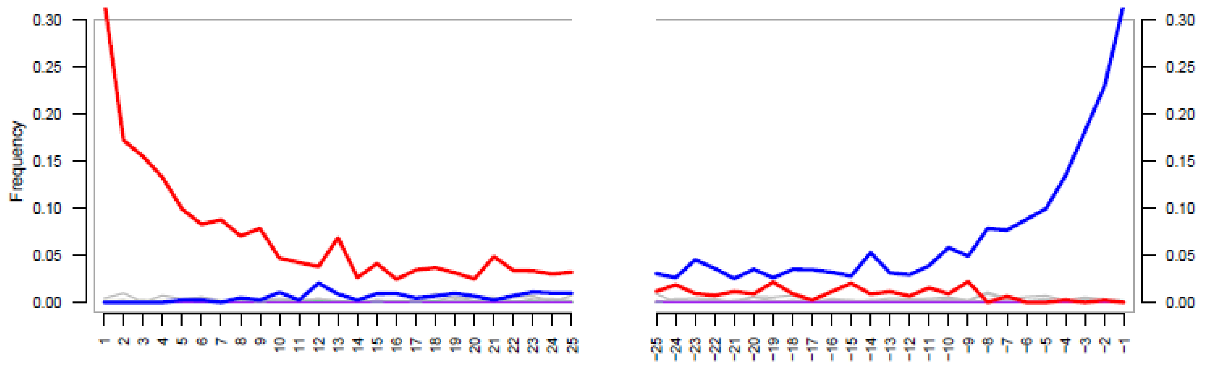

O. poz257

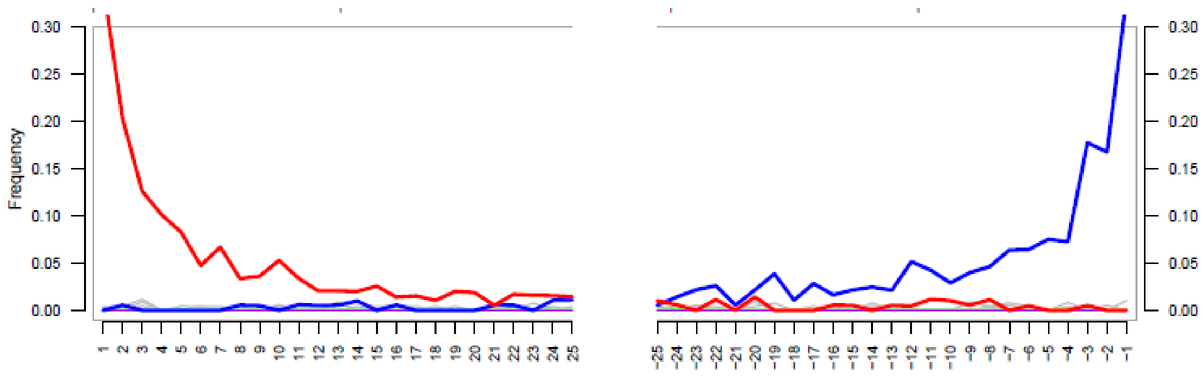

P. poz286

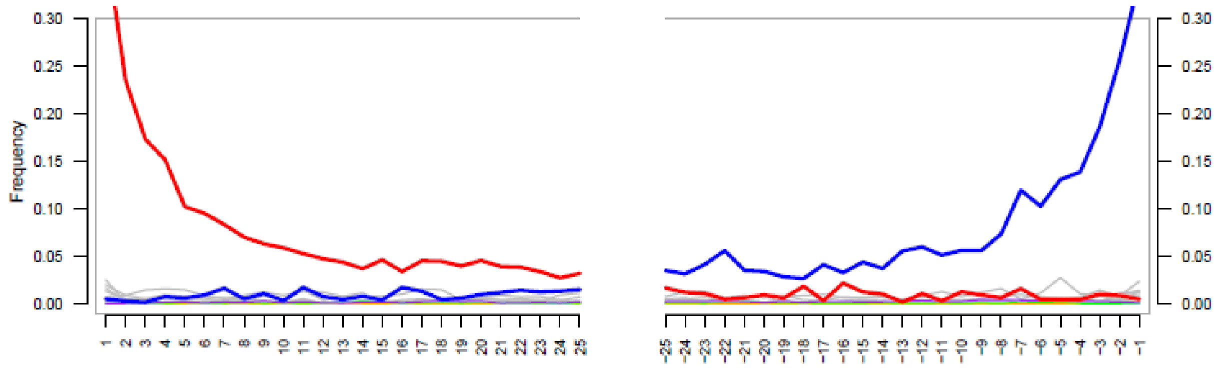

Q. poz279

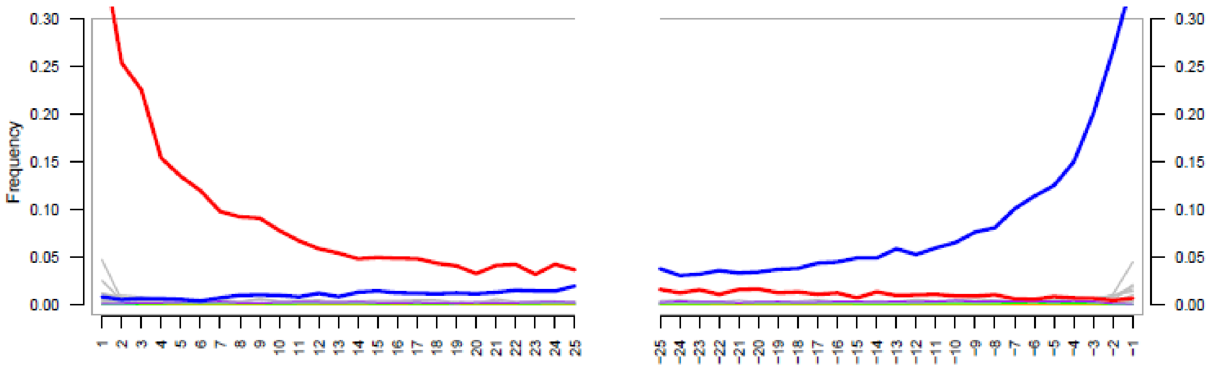

R. poz280

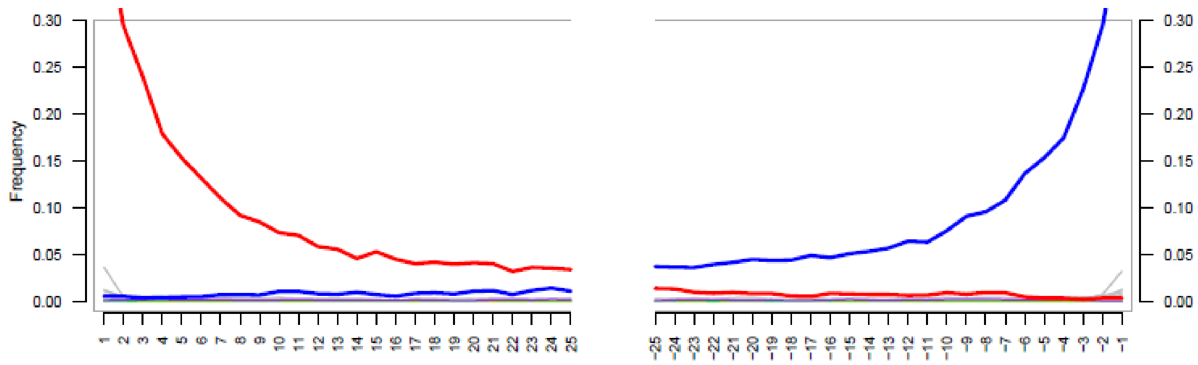

S. poz281

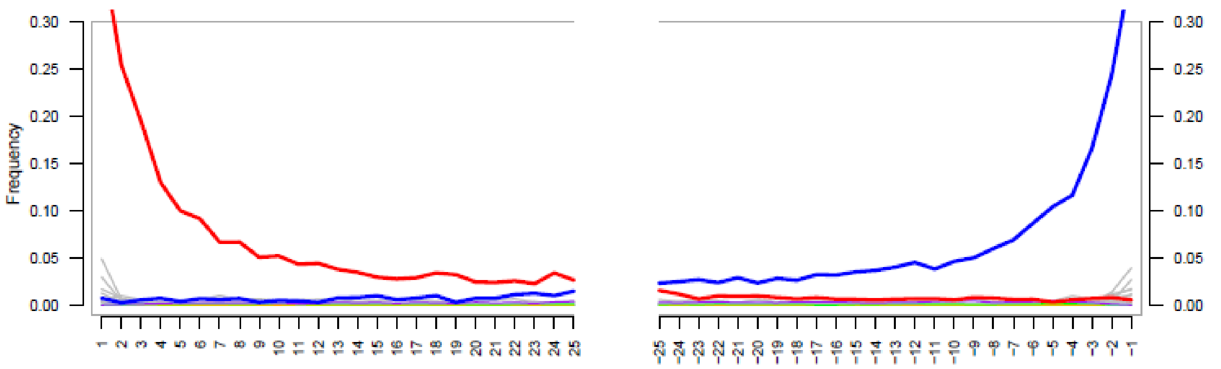

T. poz282

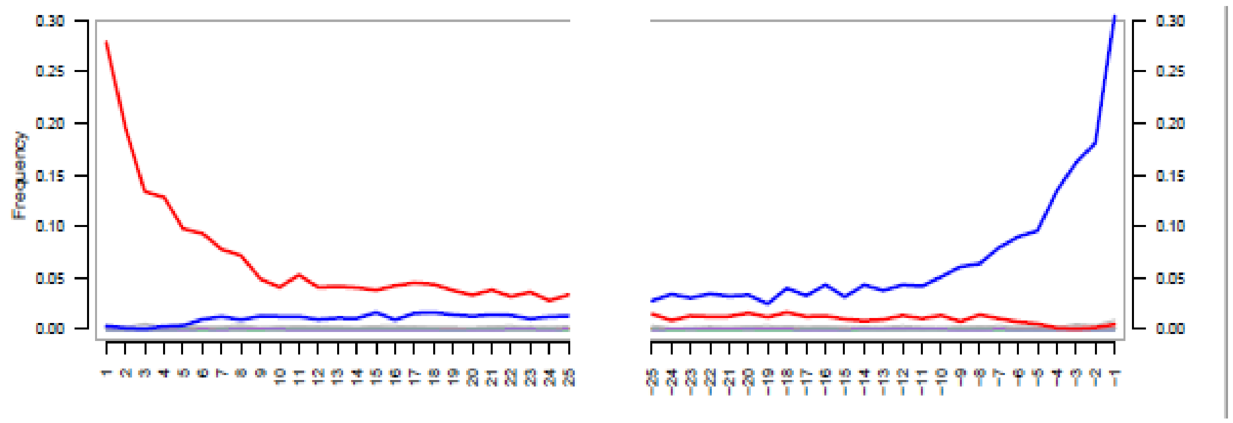

U. poz287

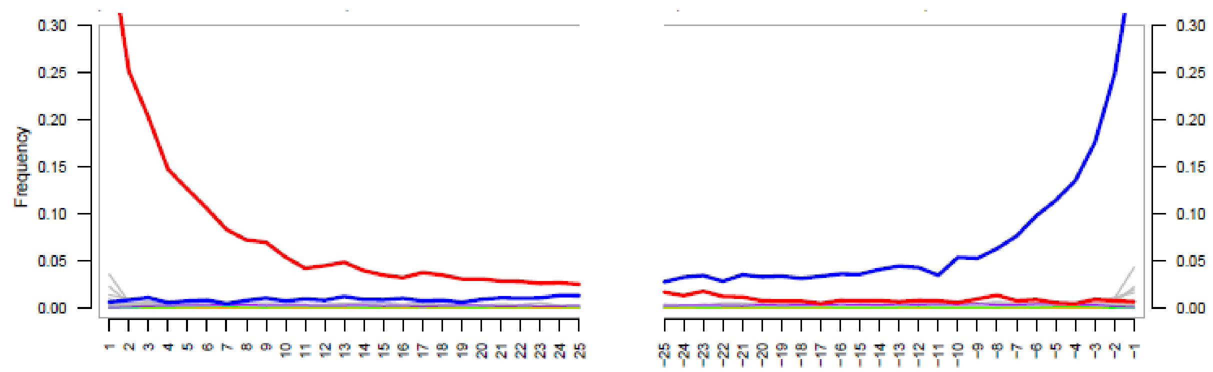

V. poz356

Supplementary Fig S26. mapDamage fragment misincorporation plots of frequency of C to T transitions (red) at the 5'ends of reads (left) and frequency of G to A transitions (blue) at the 3'ends of reads (right) for each individual.

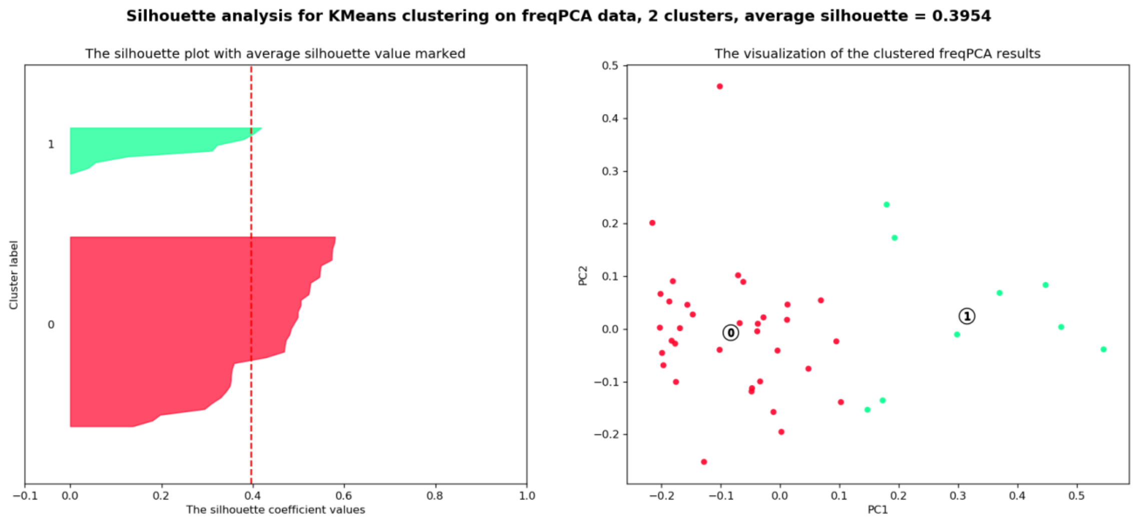
Silhouette analysis for KMeans clustering on freqPCA data, 2 clusters, average silhouette = 0.3954
The silhouette plot with average silhouette value marked
Cluster label
The silhouette coefficient values
The visualization of the clustered freqPCA results
PC1
PC2

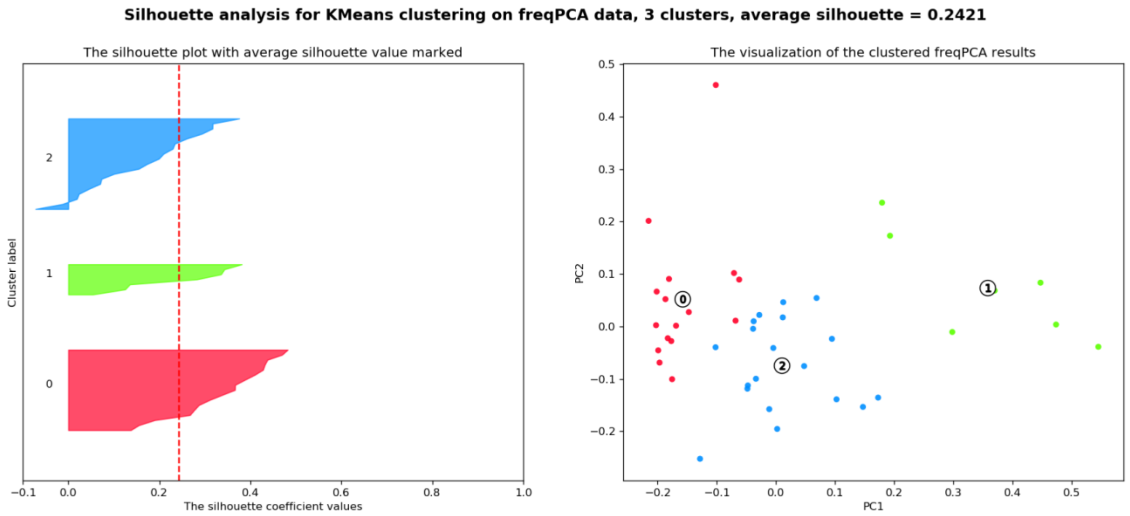
Silhouette analysis for KMeans clustering on freqPCA data, 3 clusters, average silhouette = 0.2421
The silhouette plot with average silhouette value marked
Cluster label
The silhouette coefficient values
The visualization of the clustered freqPCA results
PC1
PC2

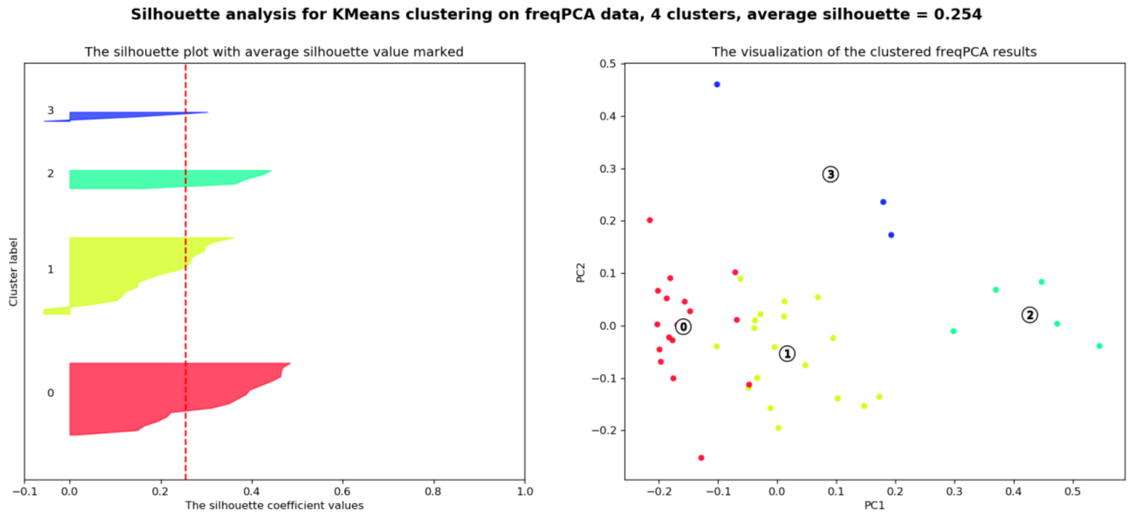
Silhouette analysis for KMeans clustering on freqPCA data, 4 clusters, average silhouette = 0.254
The silhouette plot with average silhouette value marked
Cluster label
The silhouette coefficient values
The visualization of the clustered freqPCA results
PC1
PC2

Silhouette analysis for KMeans clustering on freqPCA data, 5 clusters, average silhouette = 0.2608

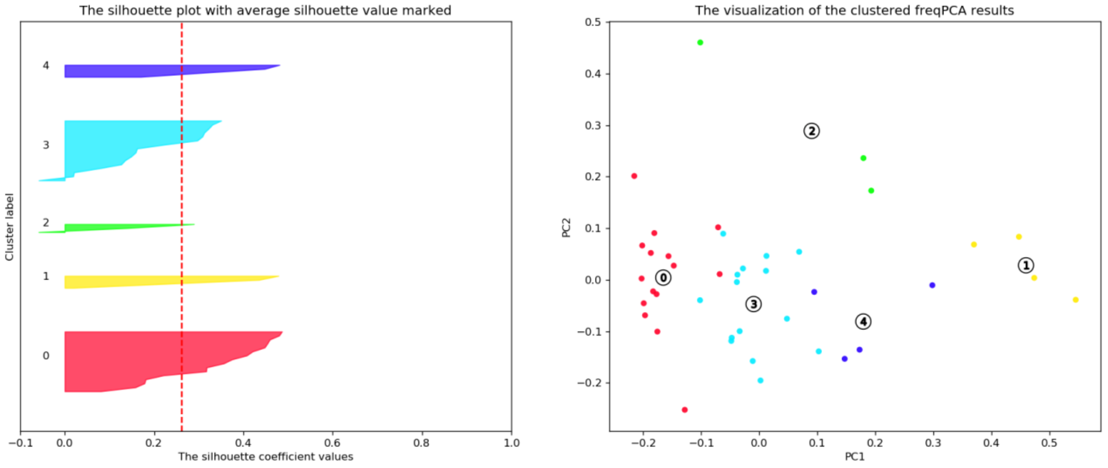

Silhouette analysis for KMeans clustering on freqPCA data, 6 clusters, average silhouette = 0.2521

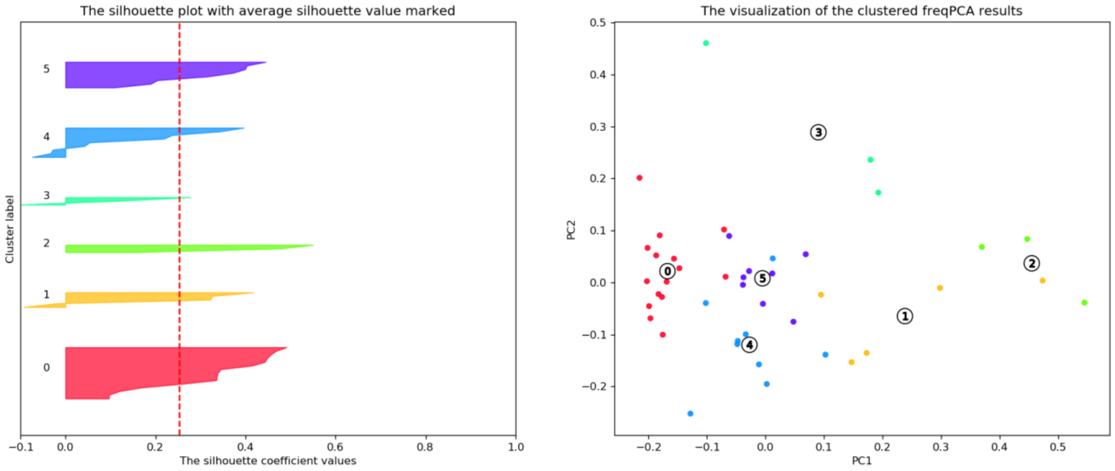

Silhouette analysis for KMeans clustering on freqPCA data, 7 clusters, average silhouette = 0.2539

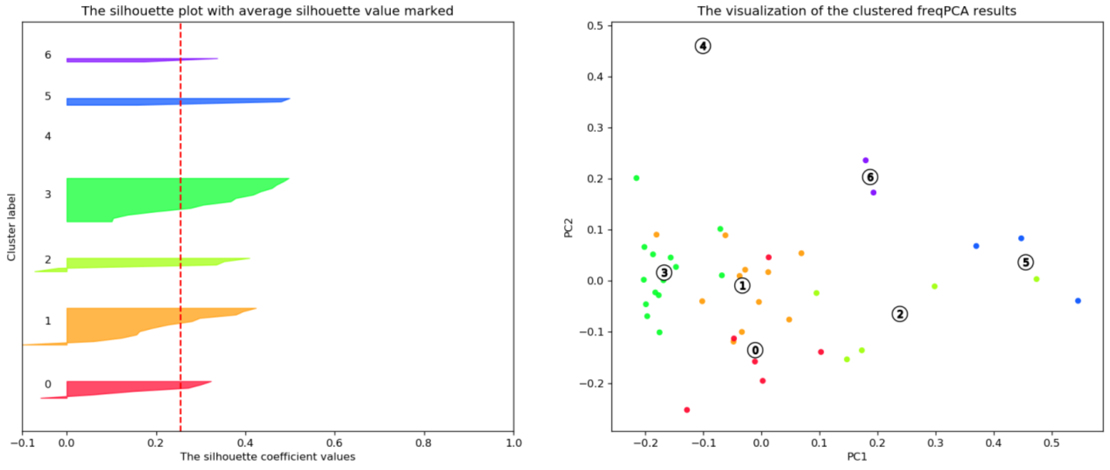

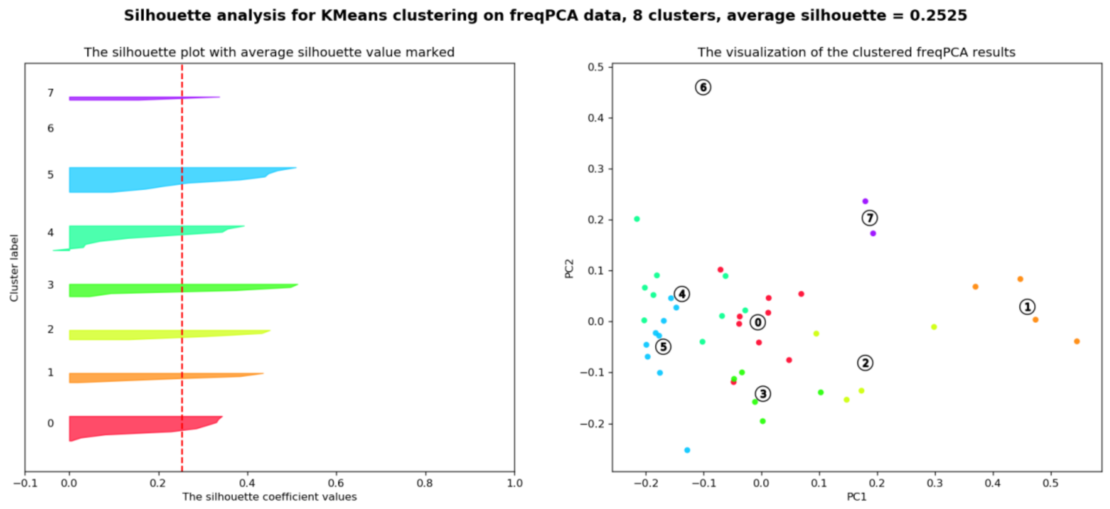

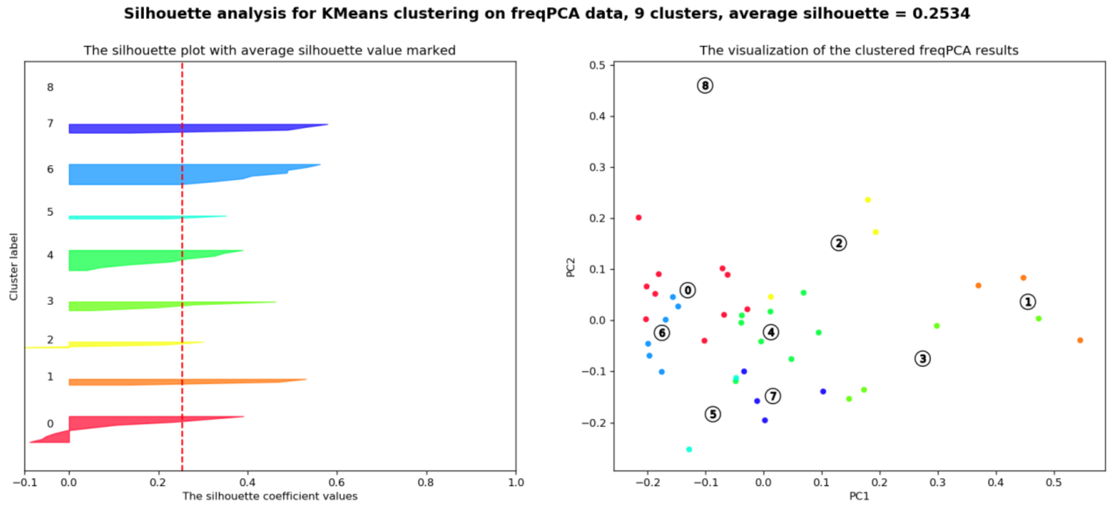

Supplementary Fig S27. K-means variants for PCA.

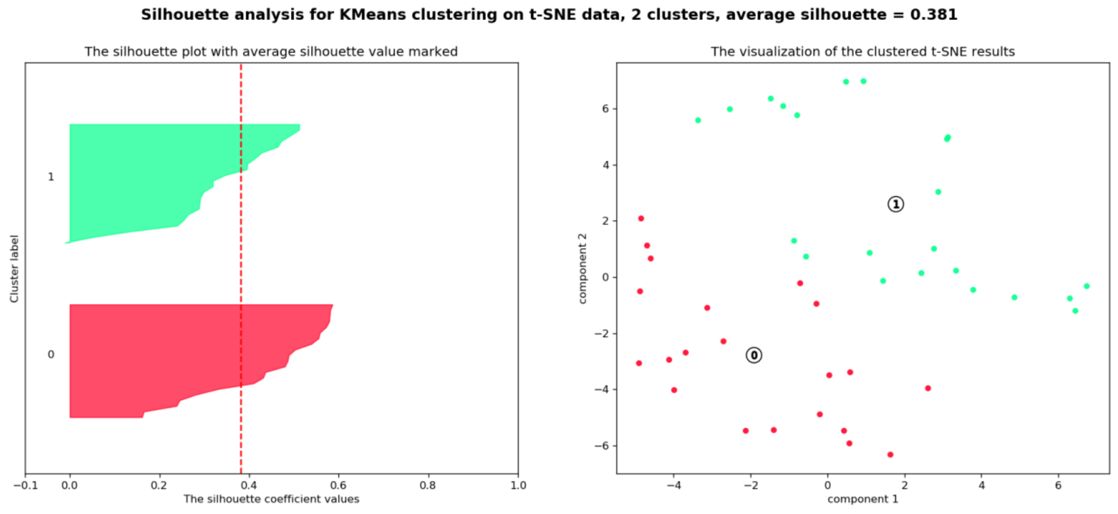

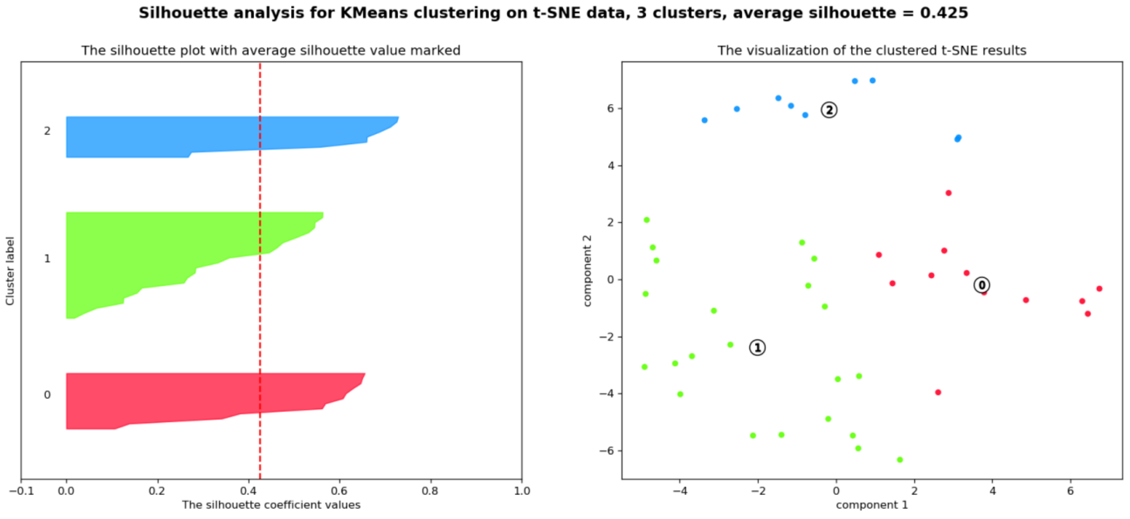
Silhouette analysis for KMeans clustering on t-SNE data, 3 clusters, average silhouette = 0.425
The silhouette plot with average silhouette value marked
Cluster label
The silhouette coefficient values
The visualization of the clustered t-SNE results
component 2
component 1

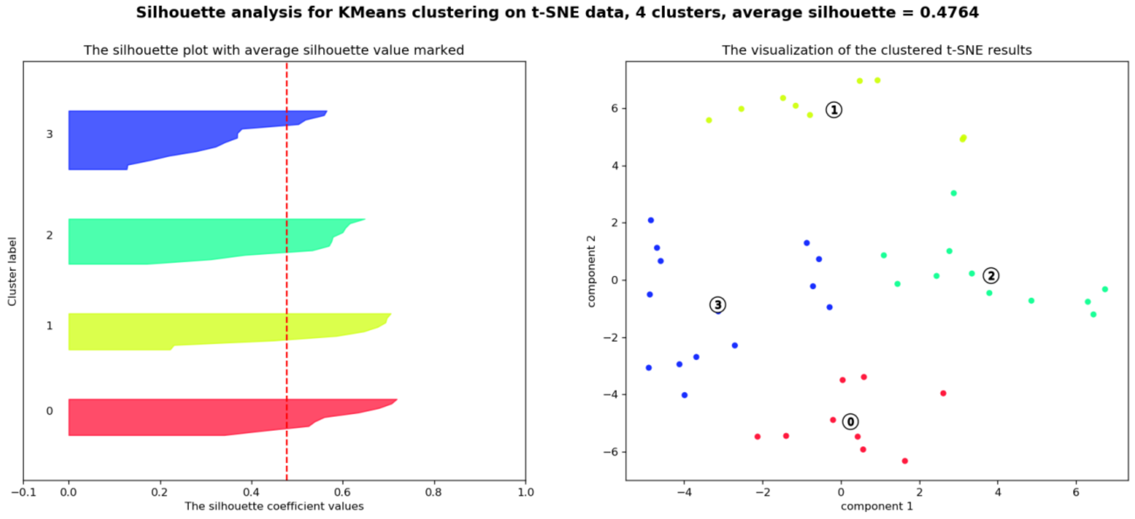
Silhouette analysis for KMeans clustering on t-SNE data, 4 clusters, average silhouette = 0.4764
The silhouette plot with average silhouette value marked
Cluster label
The silhouette coefficient values
The visualization of the clustered t-SNE results
component 2
component 1

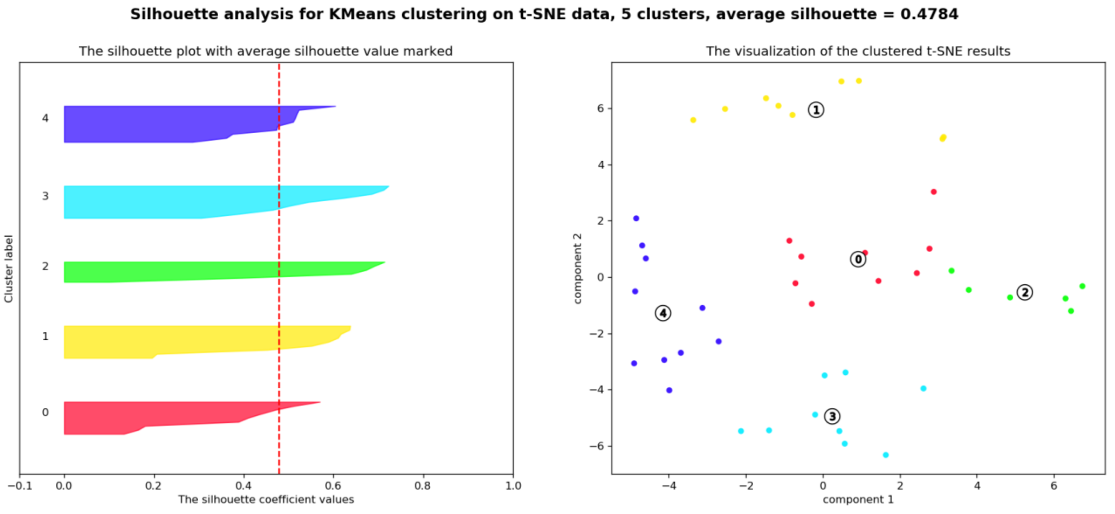
Silhouette analysis for KMeans clustering on t-SNE data, 5 clusters, average silhouette = 0.4784
The silhouette plot with average silhouette value marked
Cluster label
The silhouette coefficient values
The visualization of the clustered t-SNE results
component 2
component 1

Silhouette analysis for KMeans clustering on t-SNE data, 6 clusters, average silhouette = 0.4983

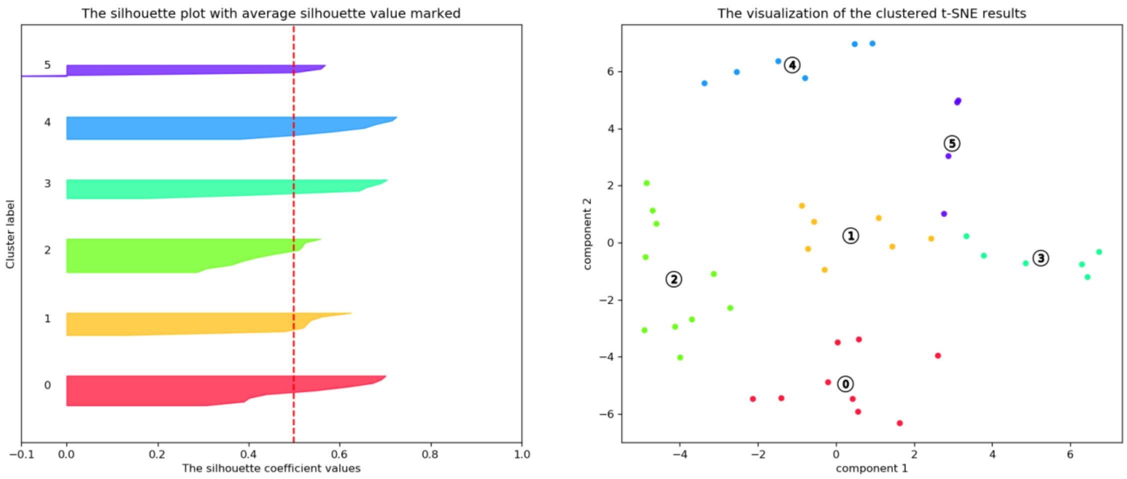

Silhouette analysis for KMeans clustering on t-SNE data, 7 clusters, average silhouette = 0.5158

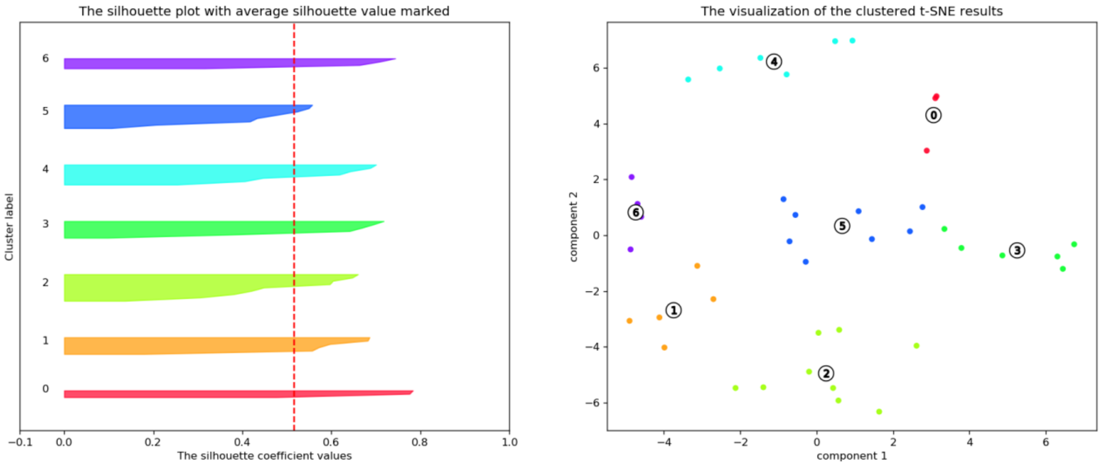

Silhouette analysis for KMeans clustering on t-SNE data, 8 clusters, average silhouette = 0.5223

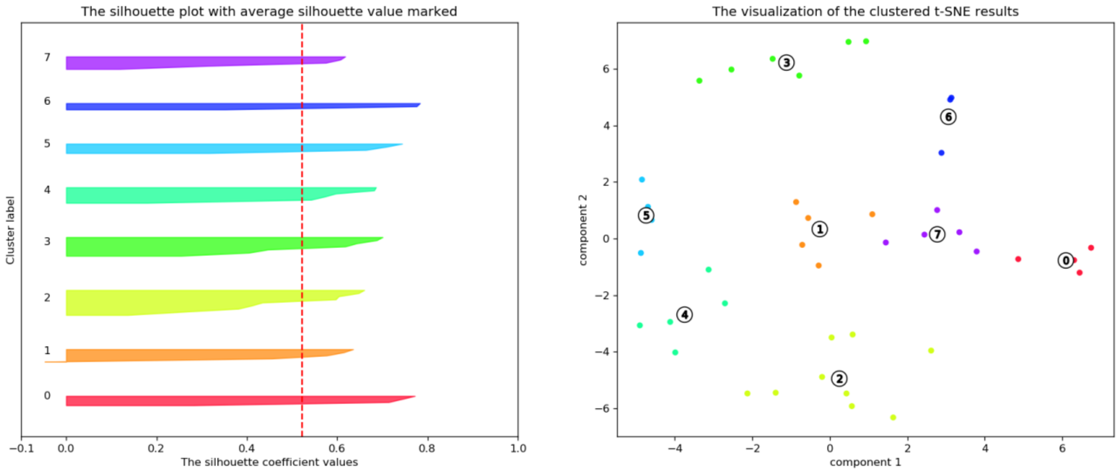

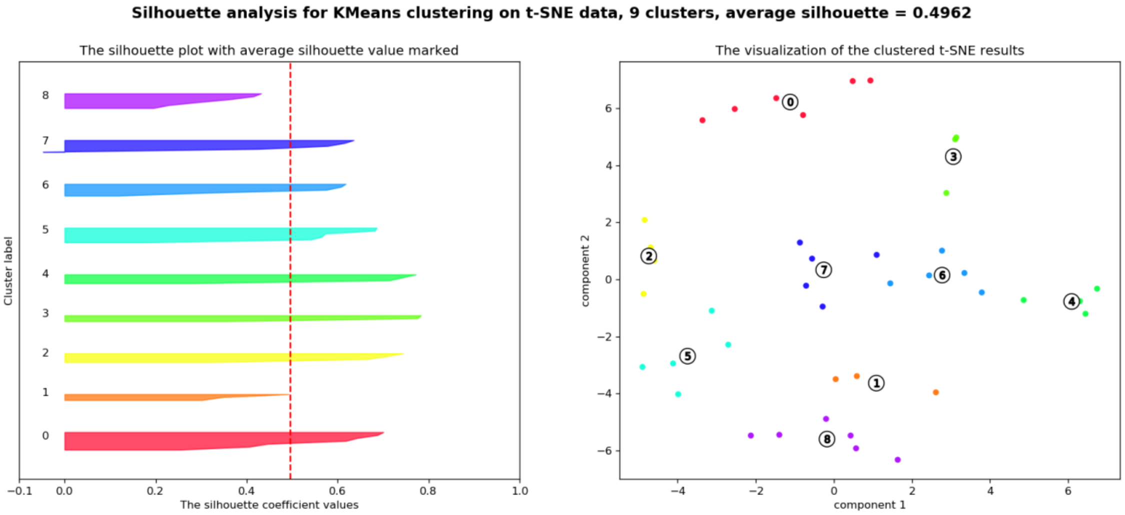

Supplementary Fig S28. K-means variants for tSNE.
